# Consequences of Peptide Macrocyclization Revealed by Virus-Inspired β‑Hairpin Mimetics

**DOI:** 10.1021/acschembio.5c00834

**Published:** 2025-12-22

**Authors:** Anna L. Bula, Raitis Bobrovs, Pavel Arsenyan, Teodors Pantelejevs

**Affiliations:** Latvian Institute of Organic Synthesis, Aizkraukles 21, Riga LV-1006, Latvia

## Abstract

Mimicry of protein secondary structure elements, such as α-helices and β-sheets, using conformationally constrained peptide macrocycles, can be utilized to disrupt native protein–protein and protein-nucleic acid interactions. Although α-helical stapled peptides have been extensively studied as pharmacological probes, the application of β-sheet and β-hairpin mimetics remains comparatively limited. Less is known about the structural and biophysical consequences of β-hairpin macrocyclization in the context of target binding. In this work, we use a poxvirus immune antagonist protein 018 as a template for the structure-based design of β-hairpin mimetic macrocyclic peptides targeting the STAT1 transcription factor. We demonstrate that successive orthogonal cyclizations have additive effects on the thermodynamic and kinetic properties of peptide binding, most notably slowing the dissociation from the target. We elucidate the structural and dynamic consequences of interstrand and head-to-tail cross-linking and propose a kinetic model explaining the gains in target residence. Finally, we highlight the pharmacological potential of these peptides by competitive inhibition of STAT1 binding to its cognate interferon receptor docking site. These data suggest that β-hairpin macrocyclization may represent a general strategy to extend target engagement, with implications for peptidic probe design.

## Graphical abstract

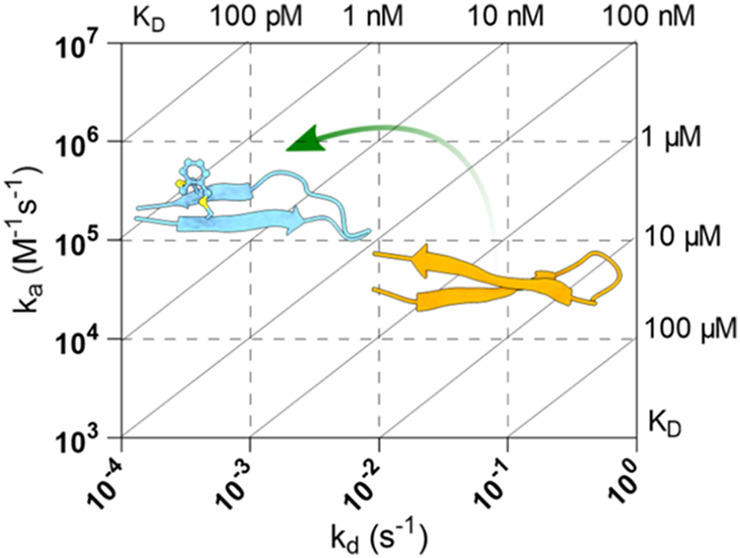

## Introduction

Protein–protein interactions (PPIs) are critical to all physiological processes, and their pharmacological modulation is a widely used therapeutic approach across many disease areas. PPIs are structurally diverse, including large complementary surfaces, as well as smaller individual secondary structure elements or linear motifs.^1^ Targeting PPIs with small molecules can be challenging due to the physicochemical properties of the involved molecular interfaces, which often lack deep, functionally relevant cavities with sufficient hydrophobic character.

Mimicry of native secondary structure elements using peptides or peptidomimetics has been applied extensively to target PPIs. Macrocyclization can further stabilize these templates and improve the affinity, proteolytic stability, and permeability. Numerous chemical strategies have been developed to stabilize α-helical peptides through side-chain stapling, with some entering clinical trials.^2–5^ β-Hairpin and β-sheet-like motifs, on the other hand, have seen considerably less application toward targeting PPIs. β-hairpins have been stabilized using turn mimetics and backbone modifications, as well as macrocylization approaches involving head-to-tail and side-chain interstrand cross-links.^6–9^ A combination of orthogonal cyclization approaches was employed in the preparation of bicyclic β-sheet mimetics as Wnt-pathway inhibitors, achieving comparable affinities to the much larger protein template.^10^ Most of these studies rely on circular dichroism or NMR experiments to demonstrate that macrocyclic and hairpin-promoting modifications increase the β-sheet character of peptides in solution. Less is known about how β-hairpin macrocyclization impacts target binding thermodynamics and kinetics, particularly when multiple constraints are combined. The source of the affinity gains observed in some of these molecules is unclear. Structural preorganization of macrocycles may lead to higher conformational selection of bound-like state, thus increasing the binding association rate and reducing the entropic penalty of binding.

In this work, we report the structure-based design of macrocyclic β-hairpin mimetic inhibitors of STAT1 transcription factor. We use a poxvirus immune antagonist protein 018 as a β-hairpin template into which we introduce structurally diverse head-to-tail and side-chain interstrand cross-links. Sequential cyclization steps lead to additive affinity gains over those of the linear peptide. We set out to extensively characterize the binding of these peptides using biophysical, structural, and molecular dynamics approaches, uncover the thermodynamic and kinetic consequences of these modifications, and highlight the determinants of improved affinity. Using these data, we propose a kinetic model that underpins the affinity gains of cyclic β-hairpin macrocycles.

## Results

### Structure-Based Design of STAT1-Binding Macrocyclic Peptides Based on a Viral Template

Viruses can hijack the host’s molecular signaling pathways to escape recognition by the immune system. Poxvirus protein 018 is overexpressed during the early stages of infection, binds STAT1 and blocks interferon-mediated innate immune response.^11^ A previously published crystal structure of the 20-residue minimal binding peptide from vaccinia virus 018 revealed that it forms an extensive β-hairpin at the SH2 domain of STAT1. Its binding mode overlaps with the site for interferon receptor phosphotyrosine and pSTAT1 self-dimerization interface, but does not interact with the pTyr pocket (Figure 1A).^11^ The β-hairpin formed by the peptide augments the central β-sheet of the SH2 domain in a parallel fashion, forming a four hydrogen bond network between the backbones of the 018 N-terminal strand and STAT1 SH2 βD strand.

**Figure 1:**
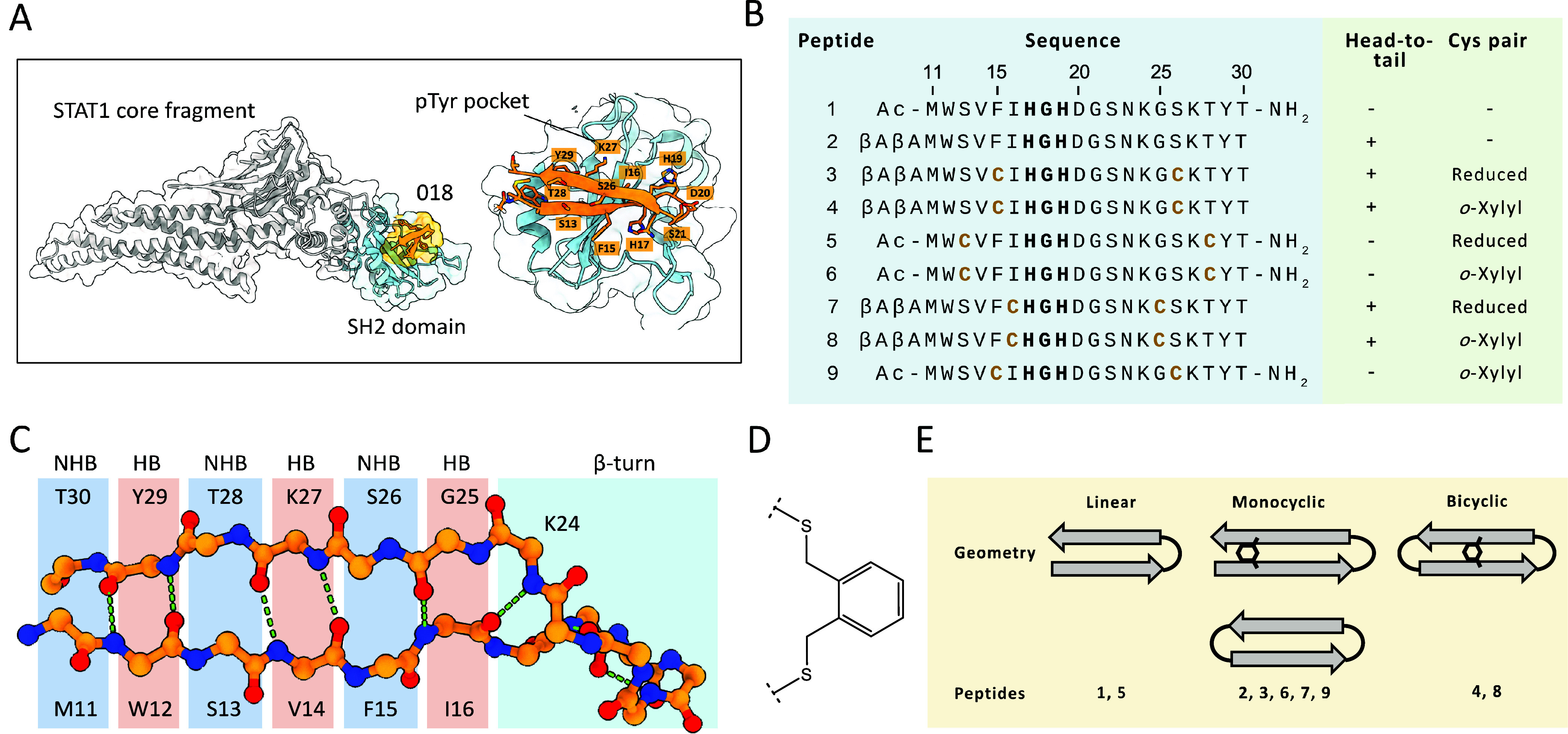
Design of STAT1-binding β-hairpin mimetic peptides based on the poxvirus 018 protein. (A) Previously published X-ray crystallographic structure of 018 minimal fragment (aa 11–30, orange, PDB: 7NUF) binding to the SH2 domain (aa 560–670, cyan) of STAT1 core fragment (gray). (B) Peptide macrocycles prepared in this work. Cysteine mutations introduced for interstrand cross-linking are highlighted in yellow. Cyclization modifications are provided on the left. βA: β-alanine. (C) Hydrogen-bonded (HB) and non-hydrogen-bonded (NHB) residue pairs within the backbone of 018. (D) Cysteine interstrand cross-linking using *o*-xylyl. (E) Schematic representation of the overall macrocyclization geometries explored in this work.

Additional specificity for the target is achieved through side-chain interactions, such as the conserved HxH motif (His17-Gly18-His19), which is also present in the phylogenetically unrelated Nipah virus phosphoprotein with similar immune antagonist function.^12^

Using the 018 minimal fragment as a template, we designed a series of macrocyclic peptides targeting the STAT1 transcription factor. In a structure-guided manner, we aimed to sample a variety of cyclization geometries to identify modifications that lead to significant improvements in the binding affinity (Figure 1B). The linear peptide **1** was prepared with N-acetyl and C-amide terminal modifications and corresponds to the wild-type sequence of the 018 minimal fragment. Peptides **2**, **3**, **4**, **7** and **8** all contained a head-to-tail amide link mediated by two β-alanine residues, a macrocyclization approach used previously for targeting β-catenin.^10^

The optimal nucleophilicity of the cysteine thiol renders it an attractive amino acid for cyclization using symmetric bis- and tris-electrophilic linkers.^13,14^ The linker plays a critical role in the design of cysteine-based cyclic peptides, as an inappropriate linker moiety may distort the native structure. For example, a simple disulfide cross-link has been shown to terminate the secondary structure of a parallel β-sheet mimetic peptide.^7^ Peptides **4**, **6**, **8**, and **9** each contained two cysteine substitutions with *o*-xylyl interstrand cross-links. *o*-Xylyl has been previously shown to be highly favorable for binding of β-hairpin macrocycles in a systematic screen of diverse bis-electrophilic linkers and was therefore selected for the present work (Figure 1D).^10^ Cross-linked peptides were synthesized from the reduced thiol precursors by the stepwise addition of α,α′-dibromo-*o*-xylene to ensure pseudodilution, thus preventing the formation of unwanted bis-linker products.

The antiparallel β-sheet secondary structure present in β-hairpins consists of alternating hydrogen-bonded (HB) and non-hydrogen-bonded (NHB) residue pairs, which are part of 10-atom and 14-atom backbone ring structures, respectively (Figure 1C).^15^ The side chains of these two classes of amino acid pairs are presented on opposite faces of the β-sheet and have different average C_α_-C_α_ and C_β_-C_β_ distances for the two pair types, which may lead to diverging linker preferences. *o*-Xylyl can bridge two cysteines within a single HB or NHB pair, whereas linking cysteines from opposite faces of the β-sheet would be geometrically incompatible and would disrupt the native secondary structure. In our series, peptides **4**, **6**, and **9** had cysteine cross-links introduced at NHB pairs (C15/C26 and C13/C28, respectively), whereas peptide **8** had an HB pair link (C16/C25). Cysteine pairs in the head-to-tail–linked peptides **4** and **8** were positioned midway between the native β-turn and the β-alanines to provide more uniform spacing between the cyclization motifs. Conversely, peptide **6** had cysteines introduced toward the termini because it lacked a head-to-tail link. Peptide **9** had the same cysteine substitutions as **3** and **4** but lacked head-to-tail modification. Other potential allowed pairs, such as C12/C29, were not tested, as they would disrupt critical contacts or introduce steric clashes.

To decouple the effects of cysteine mutagenesis from cross-linking, we also obtained the corresponding monocyclic and linear peptides **3**, **5**, and **7** with free cysteine thiols, which were maintained in reduced form in all experiments by the addition of the reducing agent tris­(2-carboxyethyl)­phosphine (TCEP). The peptide series thus sampled a variety of cyclization geometries, covering linear, monocyclic, and bicyclic peptides (Figure 1E).

### Entropy-Enthalpy Compensation in 018-Derived Macrocycles

To determine affinities and thermodynamic profiles of binding, we performed isothermal titration calorimetry (ITC) experiments for peptides **1**, **2**, **3**, **4**, **7**, and **8** (Figures 2A,B and S1,S2, Table S1). Due to insufficient solubility, we were unable to titrate peptides **5** and **6**, whereas peptide 9 had insufficient yield for ITC. The experiments were repeated in two different buffer systems, TBST (50 mM Tris pH 8.0, 300 mM NaCl, 0.01% Tween-20) and PBST (20 mM NaPi, 150 mM NaCl, 0.01% Tween-20), to account for possible buffer protonation enthalpy artifacts.^16^ The linear wild-type peptide **1** had a submicromolar affinity (K_D_ = 533 ± 20 nM in TBST), consistent with previously published ITC measurements utilizing a GB1-tagged recombinant 018 minimal fragment.^11^ The thermodynamic profile for peptide **1** indicates a relatively high entropic penalty (-TΔ*S*= 10.10 ± 1.48 kcal/mol in TBST)

**Figure 2:**
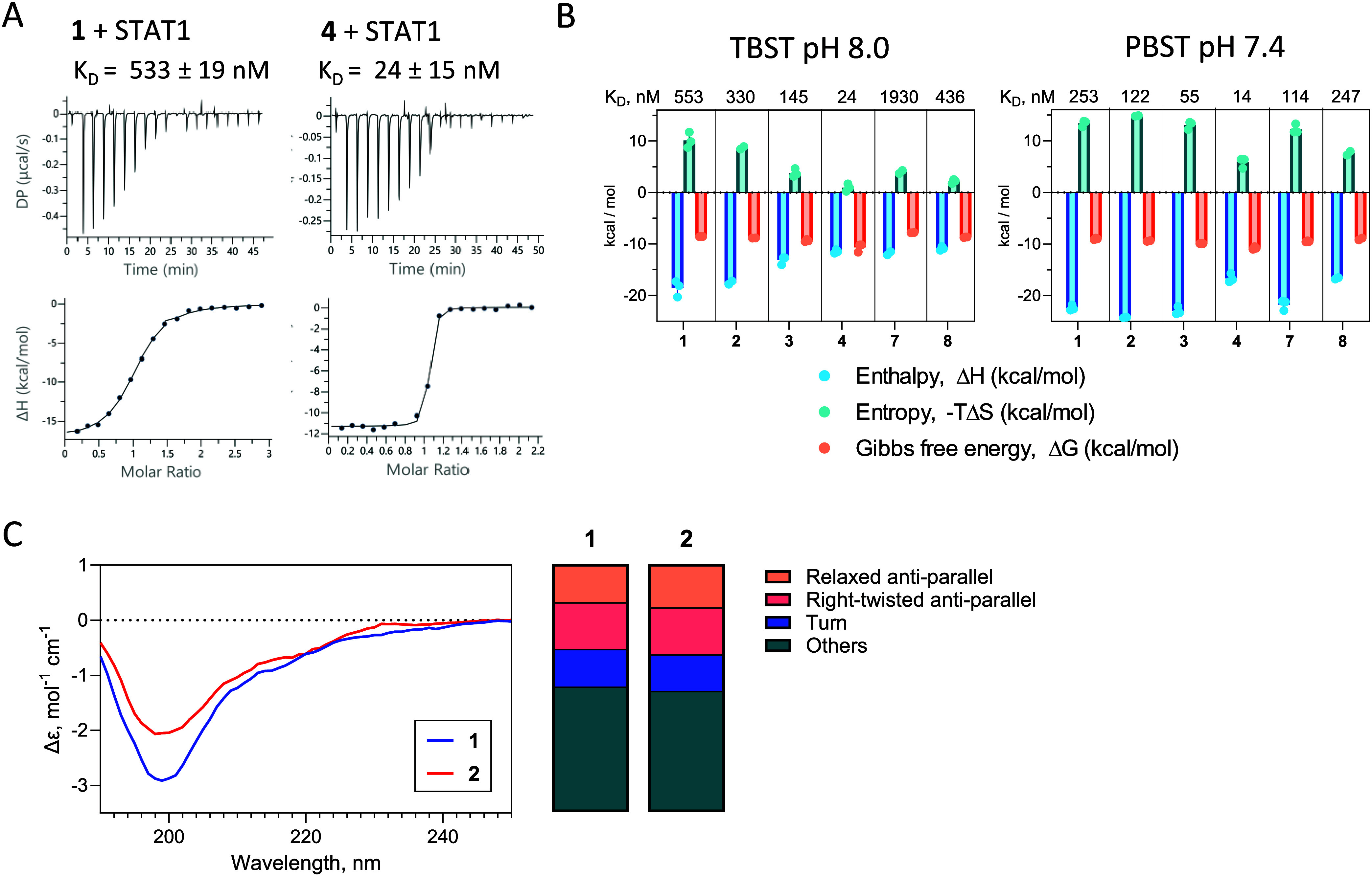
Improved affinities and entropy-enthalpy compensation are a consequence of macrocyclization in β-hairpin mimetic peptides derived from the poxvirus protein 018. (A) Representative ITC data for peptides **1** and **4**. K_D_ values are averages of three technical replicates ± SD (B) Summary of thermodynamic parameters determined by ITC for the six peptides. Measurements are shown for two different buffer systems TBST (Tris-HCl pH8.0, 300 mM NaCl, 0.01% Tween-20; left) and PBST (NaPi pH 7.4, 150 mM NaCl, 0.01% Tween-20, right). Bar charts depict average values for 2–3 technical replicates. (C) Secondary structure composition of peptides **1** and **2** in free state (left) estimated from circular dichroism spectra (right) using the BeStSel software.

that is counteracted by a high negative enthalpy (Δ*H* = −18.56 ± 1.55 kcal/mol in TBST). Head-to-tail amide-containing monocyclic peptide **2** has an approximately 2-fold improvement in affinity over **1** in both buffer systems (K_D_ = 330 ± 20 nM in TBST), with an overall similar thermodynamic profile. Notably, introduction of free cysteines at positions 15/26 in peptide **3** also has a beneficial effect on affinity, with a further 2-fold decrease in K_D_; however, the thermodynamic profiles reveal vastly different Δ*H* and -TΔ*S* values in the two buffer systems (Figure 2B). Given the much higher protonation enthalpy of Tris over phosphate, it is likely that the measurement in TBST generates artifactual Δ*H* and -TΔ*S* values for peptide **3**, whereas PBST provides a more accurate thermodynamic profile.^16^

Compared to **3**, bicyclic peptide **4** has a further 5-fold increase in affinity (K_D_ = 24 ± 15 nM in TBST), a significantly lower entropy, and a more positive enthalpy of binding in both buffer systems. Peptide pair **7**/**8** likewise demonstrates a reduction in -TΔ*S*upon linking with the *o*-xylyl moiety, however, with limited affinity gains resulting from the modification due to a counteracting decrease in enthalpy. It is possible that the HB cysteine pair introduced in peptide **8** produces less favorable thermodynamic gains than the NHB pair in peptides **4** and **6**, a structural relationship that needs to be explored in future studies with other peptide systems.

Circular dichroism (CD) spectra of peptides **1** and **2** were recorded to evaluate their secondary structure composition in the unbound state (Figure 2C). Spectra were analyzed using the BeStSel software to derive the individual contributions of different secondary structure elements.^17^ As anticipated by comparison with the crystallographic complex structure, peptides **1** and **2** lack any α-helical character and are defined by a mixture of antiparallel β-sheet, turn, and other structural elements. Interestingly, head-to-tail cyclization alone has only a marginal effect on the secondary structure composition, with a slight increase in relaxed antiparallel sheet content. This is in line with similar thermodynamic profiles of binding observed by ITC for linear peptide **1** and head-to-tail cyclic **2**. We did not analyze CD spectra for *o*-xylyl-linked peptides, as there was significant aromatic contribution to the spectrum at 230 nm which would distort secondary structure estimations.

Of the modifications introduced into the 018 template, interstrand cross-linking with the *o*-xylyl moiety has the most profound effect on the thermodynamic profiles of binding. A drop in entropy is balanced out by less negative enthalpy, leading to marginal changes in Gibbs free energy that must be fine-tuned by the optimal selection of cross-linked residues in order to achieve affinity gains. Entropy-enthalpy compensation (EEC) is a phenomenon in biomolecular recognition that has generated significant controversy regarding its artifactuality and universality.^18,19^ EEC arises when a chemical modification of a ligand results in a binding entropy difference that is offset by a simultaneous change in enthalpy or vice versa. Our data provide direct evidence of this effect in a β-hairpin peptide due to interstrand cross-linking.

### Dissociation Rate Underpins Affinity Gains in 018-Derived Macrocycles

Understanding the molecular determinants of kinetic rate constants of binding (*k*_a_, *k*_d_) can aid the design of pharmacological probes with longer target engagement, which is beneficial for drug efficacy and decreased toxicity.^20,21^ We characterized the binding kinetics of 018-derived macrocycles by using grating-coupled interferometry (GCI). We used waveRAPID, a regeneration-free kinetic binding assay that applies sequential analyte pulses of increasing duration onto a GCI biosensor surface with an immobilized target (Figures 3A, and S3,S4, Table S2).^22^ Linear peptide **1** bound STAT1 with a K_D_ of 73 nM, which is a significantly higher affinity than the one determined by ITC; however, differences of such magnitude are common when comparing orthogonal biophysical assays and different buffer conditions. The overall affinity ranking trends seen by ITC are conserved, with peptides

**Figure 3:**
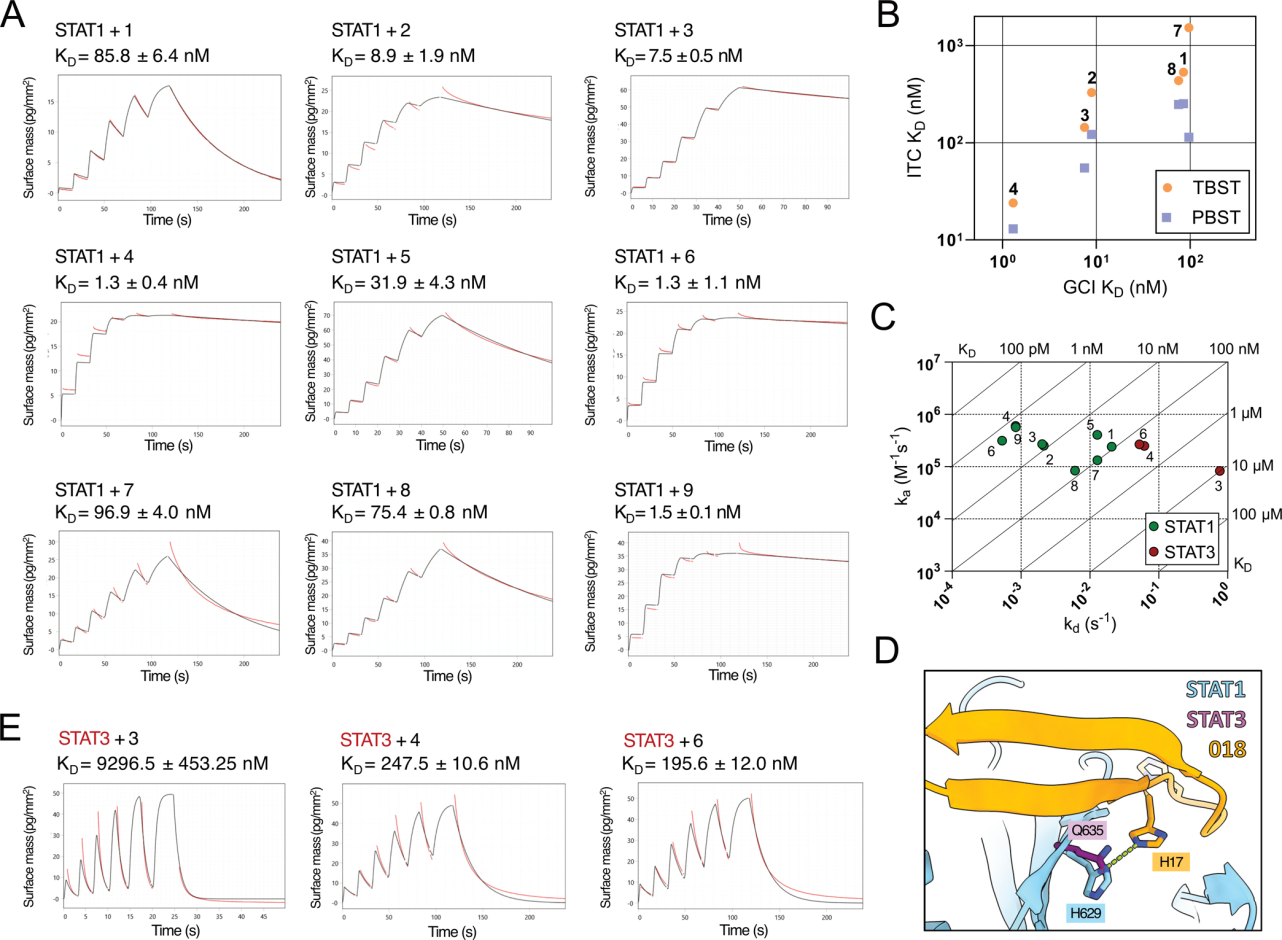
Binding kinetics of 018-derived peptides determined by grating-coupled interferometry (GCI) waveRAPID experiments. (A) Representative sensorgrams for the waveRAPID kinetics experiments with STAT1 immobilized on the 4PCH sensor chip surface. Pulses of peptide solutions were applied at increasing injection durations. Provided K_D_ values are averages of 2–4 technical replicates ± SD (B) Correlation between K_D_ values determined by ITC and GCI. (C) Isoaffinity kinetic plot showing *k*_a_ and *k*_d_ values for peptides interacting with STAT1 (green) and STAT3 (red). Values are averages of 2–4 technical replicates. (D) Interaction between 018 His17 and STAT1 H629 (light blue) compared to STAT3 Q635 (purple). (E) Sensorgrams for peptide binding to STAT3.

**1** to **4** demonstrating incremental affinity increase resulting from macrocyclization (Figure 3A,B). Enabled by the lower solubility requirements of GCI, we were able to test the peptide pair **5/6**, which also has a significant increase in affinity when cross-linked with *o*-xylyl (K_D_ = 1.3 ± 1.1 nM for peptide **6**). ITC trends are also recapitulated by peptides **7** and **8**, both of which have poorer affinities than their counterparts and display only minor affinity gains from *o*-xylyl cyclization.

The dissociation rate constant, *k*_d_, is the kinetic parameter that benefits most from macrocyclization in our peptide series, as observed in an isoaffinity kinetic plot (Figure 3C). Remarkably, both head-to-tail amide and interstrand cross-links lead to slower dissociation from STAT1. One could anticipate a cyclic β-hairpin peptide with fewer conformational degrees of freedom to be preorganized and resemble the bound state, therefore increasing the association rate constant *k*_a_ by promoting ligand conformational selection. However, we see marginal changes in *k*_a_ across the series, and in the case of peptide pairs **5**/**6** and **7/8**, cyclization actually diminishes it. An additive slowing in dissociation is observed for peptides 1 to 4, suggesting that increasing the degree of cyclization through orthogonal linkages may be a powerful approach to achieve higher affinity and longer target residence. However, peptide 9, which lacks the β-Ala-β-Ala head-to-tail link, has a comparable affinity and kinetic profile to peptide 4, pointing toward a more complex relationship between degree of cyclization and binding kinetics.

018 binds STAT1 through a β-sheet augmentation mechanism, forming an extensive hydrogen-bonding network with the β-D-strand of the STAT1 SH2 domain. In such PPIs, the bulk of the binding energy contribution typically stems from backbone interactions, whereas binding specificity is fine-tuned by side-chain contacts.^1^ Previously, the specificity of 018 to STAT1 over STAT3, another STAT family member, has been mapped to the highly directional hydrogen bond between the imidazoles of 018 H17 and STAT1 H629, which corresponds to Q635 in STAT3 (Figure 3D). We evaluated the binding kinetics of peptides to full-length STAT3 using GCI. For peptides **1**, **2**, **5**, **7**, and **8**, STAT3 binding was not detected, likely due to K_D_ being outside of the assay dynamic range. Cyclic peptides **4** and **6** both had detectable midnanomolar affinities, whereas the binding of **3** was more than an order of magnitude weaker. This suggests that β-hairpin cyclization can partially compensate for the loss of specific contacts in the homologous STAT family protein. Target affinity gains may therefore arise at the expense of selectivity.

### Interstrand Cysteine Cross-Linking Perturbs the Hairpin Secondary Structure

Atomic-level details of peptide macrocyclization can help explain the observed biophysical trends and guide future design choices. We cocrystallized the complex of monocyclic peptide **6** with a previously designed crystallographic construct of STAT1 core domain (residues 132–684) bearing entropy reduction and loop deletion mutations (Δ183–190, H182A, E393A, E394A).^11^ Crystallization attempts with other STAT1/peptide complexes did not yield diffracting crystals. We obtained diffraction data sets for two different crystal forms (Table S3). In the two structures, electron density of **6** is defined for all 20 amino acids of the peptide, which has an overall binding mode similar to that of the 018 minimal fragment (Figure 4A,B).

**Figure 4:**
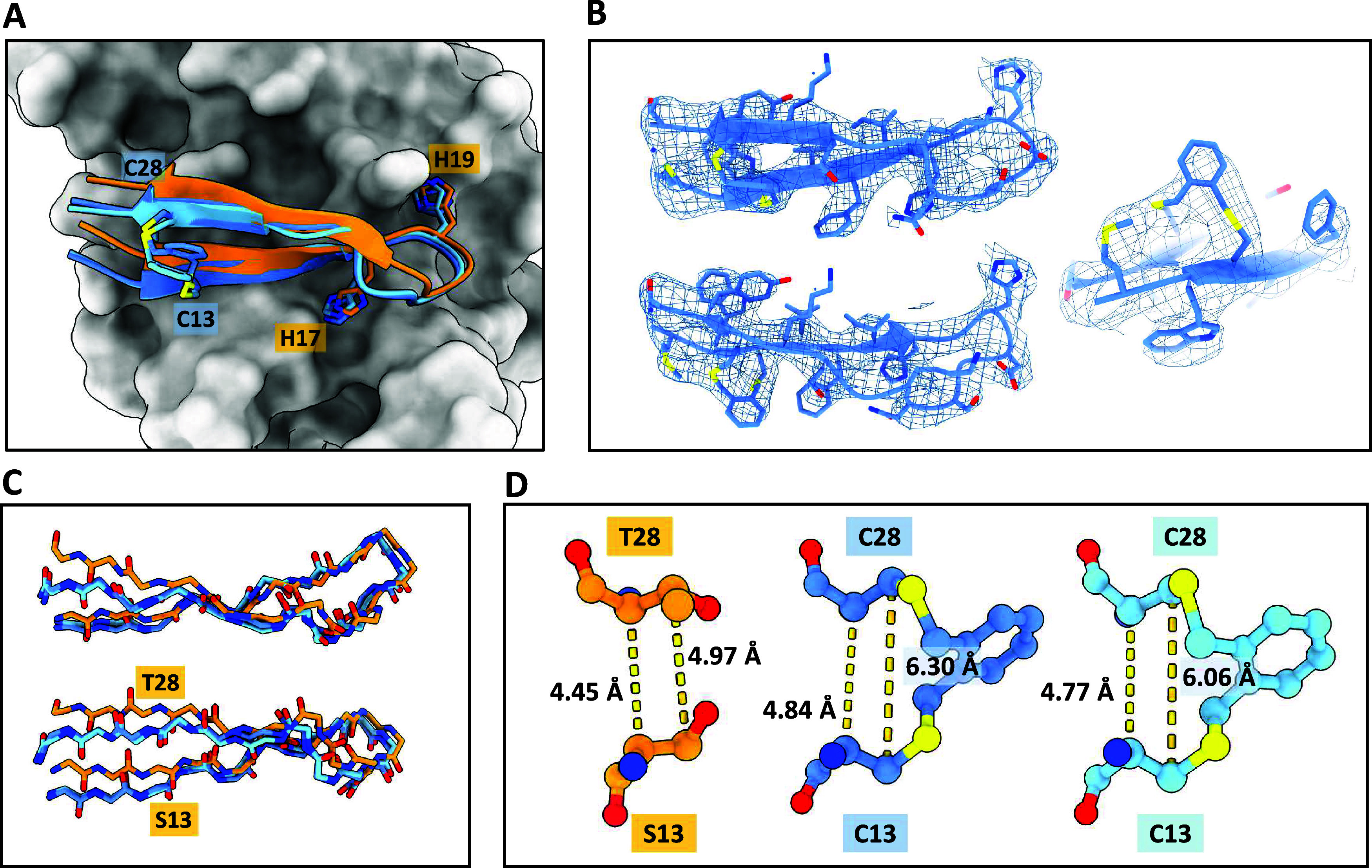
X-ray crystallographic structures of the STAT1:**6** complexes (PDB: 9IFX and 9IGA, presented in blue) compared to the linear 018 minimal fragment (orange). (A) Superposition of peptide binding modes based on the STAT1 SH2 domain residues 570–670 to account for interdomain flexibility. (B) Weighted 2mFo-DFc electron density map for peptide **6** at σ = 1.0. (C) Backbone movements observed for macrocyclic peptide **6** relative to linear 018. (D) Close-up depiction of the interstrand cross-link with C_α_-C_α_ and C_β_-C_β_ distances provided.

The *o*-xylyl moiety that connects the two cysteines is clearly resolved, suggesting limited flexibility in the interstrand cross-link, likely because of the rigid, planar four-carbon connection between the sulfurs. The linker points out toward the solvent and does not form any new contacts with STAT1. The plane of the linker phenyl ring is parallel to the direction of the peptide strands. The intra- and intermolecular hydrogen-bonding network of the peptide backbone is maintained; however, hairpin topology is perturbed significantly by the introduction of the linker (Figure 4A,C). In both crystal forms of the STAT1:**6** complex, there is significant movement of the peptide regions that are N-terminal and C-terminal to the two cysteines, compared to linear 018, with a decrease in the right-handed twist of the hairpin (Figure 4C). The central part of the peptide (Val14-Lys26), including the conserved HxH hot-spot motif, is largely unaffected by the cross-link. These binding topology changes are likely not crystallization artifacts, as crystal contacts are markedly different for the two structures. The cross-linked cysteines display a significant increase in C_α_-C_α_ and C_β_-C_β_ distances in peptide **6** relative to 018 minimal fragment (Figure 4D). Such distances are necessary to accommodate the angle between the methylene arms of the linker and are the likely reason for the rearrangement of the hairpin geometry. Even though we were not able to evaluate peptide **6** by ITC, it is tempting to explain the decreased binding enthalpies of the other *o*-xylyl-linked peptides as a direct consequence of similar secondary structure distortion.

### Integrated Model for Macrocyclic β-Hairpin Binding

Two main biophysical trends emerge in our peptide series. First, macrocyclization can reduce entropic penalty (-TΔS), while making binding enthalpy more positive, and second, macrocyclization slows down dissociation, whereas association is less affected. Transition state theory posits that dissociation can be slowed down either by stabilization of the bound ground state or by destabilization of the binding transition state.^23^ In the STAT1 GCI data (Figure 3E), we observe no correlation between *k*_d_ and *k*_a_ (*R* ^2^ = 0.09), whereas *k*_d_ with K_D_ do correlate (*R* ^2^ = 0.63), which is indicative of bound ground state stabilization relative to the unbound state, as opposed to transition state destabilization.

Our crystal structures suggest that the decreased binding enthalpy in *o*-xylyl-modified peptides stems from the suboptimal binding mode induced by the cross-link. It is therefore intriguing that ligands making weaker enthalpic contacts, which are mostly electrostatic in nature, show a decreased propensity to dissociate from the protein. We propose a kinetic model in which the linear peptides can form a partially dissociated intermediate state characterized by the opening of the C-terminal strand, resulting in fewer contacts with the protein (Figure 5A). This high-energy intermediate complex can then dissociate fully from the protein. Such a pathway would provide a decreased energy barrier for linear peptide dissociation compared to the cyclic peptides, in which the strands remain attached.

**Figure 5:**
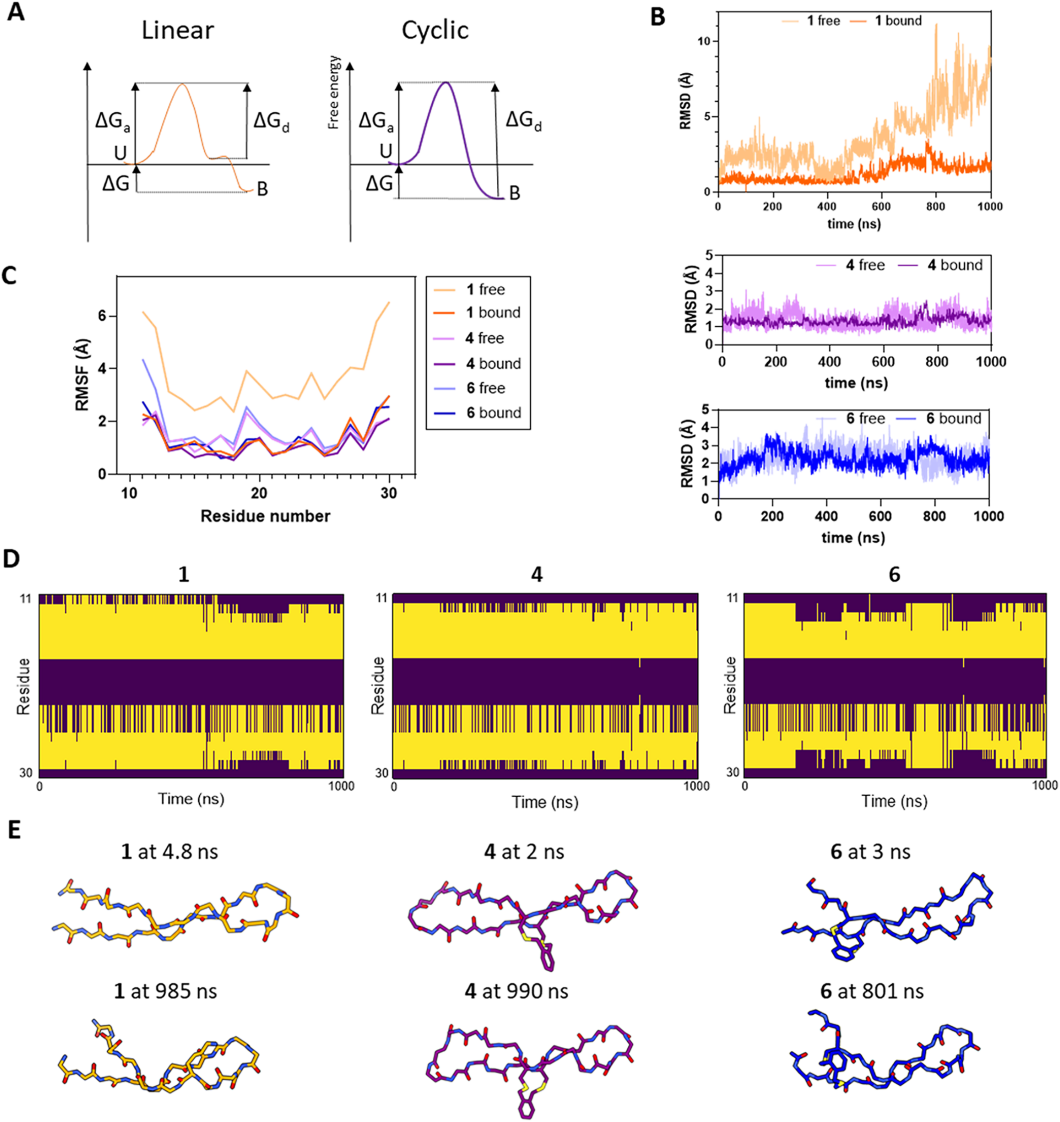
Conformational mobility of peptides **1**, **4**, and **6** in free or bound states, analyzed by all-atom molecular dynamics (MD) simulations. (A) Kinetic model for the dissociation of linear and cyclic β-hairpin peptides, U – unbound, B – bound. (B) Root mean square deviation (RMSD) values for backbone heavy atom positions relative to frame 1 over simulation time. (C) Per-residue backbone heavy atom root-mean-square fluctuations (RMSF) values. (D) Per-residue β-sheet propensity over the simulation lifetime (yellow - β-sheet formed). (E) Representative snapshots from the 1 μs simulation highlighting the partial opening of the C-terminal strand in peptides **1** and **6**.

To test this hypothesis, we performed 1 μs all-atom molecular dynamics simulations of peptides **1**, **4**, and **6** alone or in complex with the STAT1 core fragment (residues 133–683). For all peptides, the energy-minimized β-hairpin conformation was used as the starting model. RMSD and per-residue RMSF analyses show the considerable structural mobility of peptide **1** in the free state, which decreases significantly when bound (Figure 1B,C). This is in line with the high entropic penalty observed in the ITC data for **1** relative to **4**, which is the least mobile peptide in both free and bound states. Per-residue RMSF analysis reveals that the terminal residues Met11-Trp12 and Tyr29-Thr30 are the most mobile parts of the three peptides in the bound state (Figure 5C). These residues are more mobile in **1** and **6** than in **4**, likely due to the former having a head-to-tail covalent link. Per-residue β-sheet content analysis indicates that the two termini experience significant secondary structure disruption in all three peptides, with **1** and **6** showing periods of prolonged opening of the three C-terminal residues (Figure 5D). These observations are indicative of a partial opening of the C-terminal strand, which can be observed by comparison of frames from the start of the simulation with later ones (Figure 5E).

Interestingly, peptide **6** is even more prone to the opening of the terminal residues than peptide **1**, despite containing an interstrand cross-link (Figure 5E). The *o*-xylyl-induced secondary structure changes observed in the crystal structures may decrease the propensity of the terminal residues to form backbone hydrogen bonds in peptide **6**. Despite this, it has the slowest dissociation rate of the whole series, as determined by GCI, indicating that the partial opening of the C-terminus is in itself not enough to promote dissociation. It is possible that complete opening of the C-terminal strand is required to promote dissociation of the peptide via a high-energy intermediate, and this would be hindered in the case of peptide **6**. Given that the time scales accessible to classical MD are far too short to capture full dissociation events (peptide 1, the most weakly bound, has a τ of 48 s), our simulations provide only an incomplete and thus tentative picture of the underlying mechanism. Nevertheless, our MD simulations point toward a strong propensity of the linear peptide to undergo partial opening of the secondary structure at the C-terminus. Such movement of the peptide chain, upon reaching a sufficient extent, may significantly lower the transition state energy barrier due to the weakening of interactions between the C-terminal strand and the protein. Future MD studies utilizing enhanced sampling of dissociation events, such as replica exchange or metadynamics, will help to substantiate the proposed kinetic model.

Thermodynamic properties of stapled peptides and other constrained macrocycles, mainly entropic penalty reduction, have been examined previously as the driving force behind improved binding, whereas kinetic changes remain obscure. The biophysical and *in silico* data presented suggest that restrained conformational dynamics may have profound effects on the binding kinetics of β-hairpin mimetics. A single interstrand cross-link in **6** increased the target residence time (τ = 1/*k*_d_) approximately 30-fold compared to the linear peptide **5**, in the absence of any new contacts with the protein. The gradual decrease in *k*_d_ in peptides **1** to **4** supports increasing the degree of cyclization as a general strategy for improving both the affinity and target residence time.

### β-Hairpin Mimetics Inhibit Interferon Receptor Phosphotyrosine Site Recognition

To be utilized as STAT1 inhibitors, the obtained peptides must disrupt the interaction between the STAT1 SH2 domain and its cognate interferon receptor phosphotyrosine site, thus preventing phosphorylation and downstream signaling. We evaluated peptides **1**, **3**, and **4** in a fluorescence polarization assay using a 12-mer pIFNGR1 peptide conjugated to fluorescein as a competition probe. All three peptides were able to displace the probe, as evidenced by a decrease in the polarization (Figure 6A). Compared to the linear peptide **1**, the monocyclic **3** and bicyclic **4** had approximately 4-fold lower IC_50_ values (4.3, 1.3, and 1.1 μM, respectively), consistent with the biophysical affinity data. We were not able to discriminate the potencies of **3** and **4**, as they were outside the dynamic range of the assay, which is limited by ligand depletion and the requirement for a high concentration of STAT1 protein (1.5 μM).

**Figure 6:**
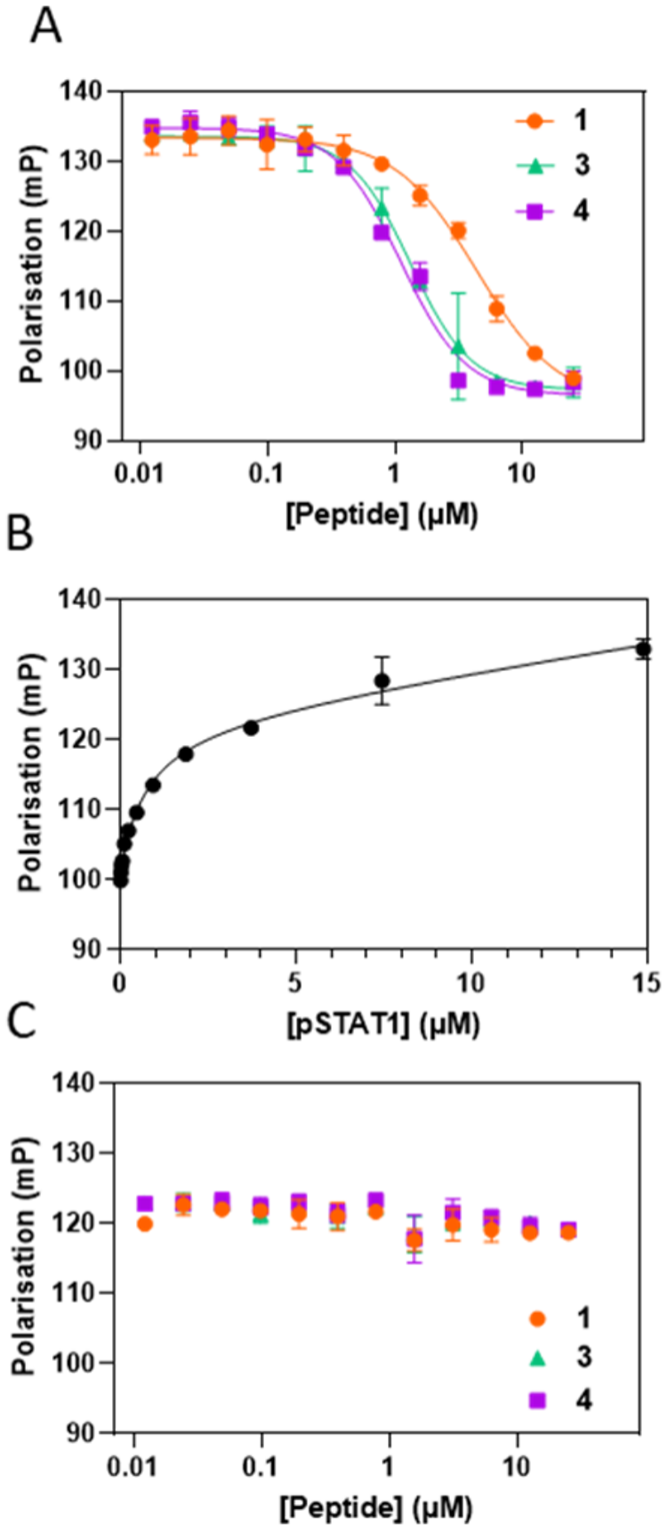
Macrocyclic β-hairpin mimetics disrupt the association between STAT1 and its receptor docking site, but not STAT1/GAS DNA complex. (A) Fluorescence polarization (FP) competition assay using a fluorescein-labeled interferon γ receptor phosphotyrosine docking site peptide. (B) Direct FP binding measurements of pSTAT1 with a GAS dsDNA oligonucleotide. (C) FP titration of peptides into preformed pSTAT1:GAS dsDNA complex.

Inhibition of the reciprocal interaction between the two protomers of phosphorylated STAT1 (pSTAT1) would likewise prevent interferon signaling by disrupting the parallel pSTAT1 dimer and formation of the transcription complex.^24^ We tested if our peptides disrupt the interaction between a pSTAT1 homodimer and the cognate γ-activated sequence (GAS) dsDNA oligonucleotide using a competition FP assay. Fluorophore-labeled GAS duplex DNA sequence (FAM-5′-GATGTATTTCCCAGAAAAGG-3′) was first titrated with pSTAT1, demonstrating a concentration-dependent increase in polarization (Figure 6B). A much lower increase was observed with an ISRE duplex DNA sequence, which preferentially binds STAT1/STAT2 heterodimers (Figure S5). The addition of peptide to the preformed STAT1/GAS complex, however, did not disrupt the interaction with DNA even at 4-fold stoichiometric excess over the protein (Figure 6C). This suggests that disassembly of an activated STAT1/DNA complex is more challenging that inhibition of receptor docking.

## Discussion

We have presented detailed thermodynamic, kinetic, and structural consequences of interstrand and head-to-tail macrocyclization in a series of β-hairpin mimetic peptides. Our findings highlight a clear rationale for using these chemical modifications to not only improve binding affinity but also significantly extend the target residence time of the peptide, a desired property for a pharmacological probe. It remains an open question whether these biophysical findings are transferable to other peptide:target systems, and future studies are needed to confirm broader applicability. The obtained X-ray crystallographic data support the application of *o*-xylyl for interstrand cross-linking, while highlighting potentially disruptive structural consequences. Future research efforts should extensively evaluate the compatibility between linker moieties and cross-link geometries, such as HB and NHB residue pairs, similarly to the rules suggested for α-helical stapled peptides.^25–27^

Interactions between the virus and host factors are an essential feature of the viral life cycle. Short linear motifs (SLiMs) in viral proteins, such as the conserved HxH motif of poxvirus 018 and Nipah virus phosphoprotein, enable fast adaptation to changing host environments via high mutational rates and structural flexibility.^28,29^ There are an estimated 40,000 mammalian viruses when host sharing is taken into account.^30^ The diversity of these combined host–virus interactomes is thus an undertapped source of structural and functional information for the development of new pharmacological probes. To the authors’ knowledge, there are only limited examples published to date of viral factor templates being converted into pharmacological probes.^31^ In this work, we achieved a remarkable improvement in affinity relative to the natural viral sequence, providing a rationale for further studies utilizing macrocyclization of viral SLiMs to target poorly druggable members of the human proteome.

STAT1 gain-of-function (STAT1 GOF) is an immune disorder characterized by an increased phosphorylation of STAT1 following interferon stimulation, leading to a broad clinical presentation, including life-threatening invasive infections.^32^ The current standard of care includes antifungals and anti-inflammatory drugs, as well as experimental treatments such as hematopoietic stem cell transportation and JAK inhibition.^33^ Pharmacological inhibition of STAT1 is a potential alternative therapeutic approach for treating STAT1 GOF. STATs are a challenging target class and most of the probes have poor pharmacological properties as they mimic the pTyr moiety.^34^ Highest-affinity peptides **4** and **6** shows superior binding to previously published STAT family targeting molecules, including clinical stage inhibitors.^35^ Future studies should focus on the optimization of peptide size, physicochemical properties, and delivery in order to obtain cell-active inhibitors with favorable metabolic and pharmacokinetic properties.

## Supplementary Material



## Notes

The Supporting Information is available free of charge on the ACS Publications Web site. Materials and methods, ITC data and fitted parameters, GCI data and fitted parameters, direct FP titrations of STAT1 with ISRE/GAS DNA, X-ray crystallographic data collection and refinement statistics, peptide analytical data, STAT3 gene sequence, materials and methods bibliography (PDF)
