# Supplementary material for: Consequences of Peptide Macrocyclization Revealed by Virus-Inspired β‑Hairpin Mimetics
Source: ACS Chem Biol. 2025 Dec 22;21(1):160–9. doi: 10.1021/acschembio.5c00834 (PMC12813973; doi:10.1021/acschembio.5c00834)
Supplement: Supplementary file 1 [file cb5c00834_si_001.pdf]

Supporting Information

Consequences of Peptide Macrocyclization Revealed by Virus-inspired β-Hairpin Mimetics

Anna L. Bula,[a] Raitis Bobrovs,[a] Pavel Arsenyan,[a] Teodors Pantelejevs*[a]

[a] A. L. Bula, R. Bobrovs, P. Arsenyan, T. Pantelejevs

Latvian Instititue of Organic Synthesis

Aizkraukles 21, LV-1006, Riga, Latvia

E-mail: teodors.pantelejevs@osi.lv

Table of contents

Supporting information ... 1

Table of contents ... 2

Experimental methods ... 3

Peptide synthesis ... 3

Cysteine cross-linking with α,α′-dibromo-o-xylene ... 3

Peptide purification ... 3

Analytical MALDI-TOF MS ... 3

Analytical HPLC ... 4

Protein concentration determination ... 4

Protein purification ... 4

Isothermal titration calorimetry (ITC) ... 5

Grating coupled interferometry (GCI) ... 6

Molecular dynamics simulations ... 6

Fluorescence polarisation (FP) assay ... 6

Results and Discussion ... 9

X-ray crystallographic data collection and refinement ... 16

Peptide analytical data ... 17

STAT3 synthetic gene sequence ... 25

Bibliography ... 27

Experimental methods

Peptide synthesis

Unless otherwise stated, all reagents were purchased from commercial suppliers and used without further purification. All peptides were initially obtained in linear or head-to-tail cyclic form from GenScript Biotech Co., Ltd. (Zhenjiang, Jiangsu Province, China) at >75% purity. Peptides were synthesised using standard Fmoc/t-Bu approaches.

Cysteine cross-linking with α,α′-dibromo-*o*-xylene

10-15 mg of cysteine pair-containing linear or head-to-tail amide cyclic peptides were dissolved in 100 mM ammonium bicarbonate / MeCN (1:1, v/v) to a concentration of 1 mM. TCEP-NaOH (0.5 M, pH 8.0, in ultrapure water) was added to a final concentration of 0.5 mM to prevent intra- and inter-molecular disulfide formation. 100 mM α,α′-dibromo-*o*-xylene linker solution in MeCN was added in 10 µl increments to the peptide solution while stirring at room temperature. Linker was added every 10 minutes to a 2:1 final molar excess to ensure pseudo-dilution and minimise formation of double-linker adducts. Reaction progress was monitored by MALD-TOF MS and quantitative conversion was observed after all the linker had been added. Remaining α,α′-dibromo-*o*-xylene was quenched with an excess of dithiothreitol (1 M, in ultrapure water), followed by acidification with TFA. We did not observe any TCEP-linker-peptide adduct formation.

Peptide purification

Peptides were purified by reversed-phase HPLC on an AKTA Pure chromatography system (Cytiva) using the following column: XBridge Prep C18, 130 Å, 10 µm, 19x250 mm (Waters #186004021). Prior application, peptide solutions or reaction mixtures were diluted in 0.1% TFA and filtered using a 0.2 µm syringe filter (Sarstedt #83.1826.001). Peptides were purified using different gradients of A (ultrapure water + 0.1% TFA) and B (MeCN + 0.1% TFA) at a flow rate of 20 ml/min. Peak fractions were pooled and TFA removal was performed by slow evaporation of acetonitrile, followed by 3 cycles of addition of 1 ml of 0.5M HCl in Et_2O in an ice bath and evaporation by 50% volume. Finally, peptides were lyophilised overnight. Based on HPLC data the purities of the obtained peptides were >95% with the exception of **2**, which we were unable to purify to such level.

Analytical MALDI-TOF MS

Peptide DMSO stocks (10-20 mM) were diluted 500x in ultrapure water containing 0.1% TFA. Peptide solutions were plated on a 384-well ground steel target plate by mixing

with 2 mg/ml α-cyano-4-hydroxycinnamic acid solution in 50% MeCN, 0.1% TFA. After drying, spectra were collected on an autoFlex Max instrument (Bruker) using positive ion reflector mode with a m/z range of 900-4500. Spectra were summed from several collections, each with 500 laser shots, 2 kHz laser frequency, 25-40% laser power. Monoisotopic mass lists were extracted using FlexAnalysis software (Bruker). Mass spectra are shown in supplementary section **Peptide analytical data**.

Analytical HPLC

Peptides were analysed on an ÄKTA Pure chromatography system (Cytiva) using an Xselect Peptide CSH C18 (130 Å, 5 µm, 4.6x150 mm) column (Waters #186007078), at a flow rate of 1 ml/min using solvent A (0.1% TFA in ultrapure water) and B (0.1% TFA in MeCN). A 5-65% solvent B gradient was used over 30 min. Absorbance was detected at 225 nm.

Protein concentration determination

All protein and peptide concentrations were determined using UV-Vis absorbance at 280 nm on a Nanodrop One instrument (ThermoFisher). Extinction coefficients were calculated using the Protparam application.[1] The *o*-xylyl modifications on cyclic peptides were assumed to have a negligible effect on absorbance at 280 nm as the peptides contained Trp and Tyr residues.

Protein purification

STAT1: full-length STAT1 (Uniprot #P42224-1) was expressed in *E. coli* using a previously published protocol.[2] *E. coli* T7 Express cells (New England Biolabs #C2566H) carrying the pEXP-NHis-STAT1 plasmid were grown in 2 l culture flasks in 2xYT medium at 37°C until an OD_{600} of 0.8. The cells were then cooled down to 18°C and overnight expression induced with 0.5 mM IPTG. Next, day, cells were harvested and resuspended in IMAC A buffer (50 mM Tris pH 8.0, 500 mM NaCl, 20 mM imidazole) supplemented with 5% glycerol. Cells were lysed using sonication and centrifuged at 48 000 xg for 45 min. Clarified supernatant was applied onto a HisTrap HP 5 ml (Cytiva #17524801) column, which was then washed with 10 CV IMAC A buffer. Bound protein was eluted with IMAC B buffer (50 mM Tris pH 8.0, 500 mM NaCl, 200 mM imidazole). Protein was concentrated using a Vivaspin 20 centrifugal concentrator (10 kDa MWCO, Sartorius #VS2002) and further purified using a Superdex 200 16/600 column (Cytiva #28989335) equilibrated with SEC buffer (20 mM HEPES pH 7.5, 300 mM NaCl, 0.5 mM TCEP).

STAT3: the gene encoding full-length STAT3 was codon optimised for expression *in E.coli* and cloned by Gibson assembly into the pEXP-MBP plasmid (addgene #112568) digested

with BamHI/HindIII enzymes (New England Biolabs # R3136S, R3104S). The gene sequence is provided in section **STAT3 synthetic gene sequence**. The plasmid was transformed into chemi-competent T7 Express *E. coli* cells (New England Biolabs #C2566H), which were then grown at 37°C in 2xYT medium in 2 l flasks to an OD_{600} of 0.8 and then cooled down to 18°C. Overnight expression was induced with 0.5 mM IPTG. Next, day, cells were harvested and resuspended in IMAC A buffer (50 mM Tris pH 8.0, 500 mM NaCl, 20 mM imidazole) supplemented with 5% glycerol. Cells were lysed using sonication and centrifuged at 4°C, 48 000 xg for 45 min. Clarified supernatant was applied onto a HisTrap 5 ml (Cytiva #17524801) column, which was then washed with 10 CV IMAC A buffer. Bound protein was eluted with IMAC B buffer (50 mM Tris pH 8.0, 500 mM NaCl, 200 mM imidazole). The sample was buffer-exchanged into TEV buffer (20 mM Tris pH 8.0, 300 mM NaCl, 0.5 mM TCEP) on a HiPrep Desalting columns (Cytiva #17508701) and supplemented with 50 µg/ml TEV protease (prepared in-house) for overnight cleavage at 4°C. Next day, the protein was purified by reverse IMAC using the same buffers as for the initial capture step. The flow-through was concentrated un purified by size-exclusion chromatography the same way as STAT1.

Isothermal titration calorimetry (ITC)

ITC experiments were performed using a PEAQ ITC instrument (Malvern Panalytical) in either TBST (50 mM Tris pH 8.0, 300 mM NaCl, 0.5 mM TCEP, 0.01% Tween-20) or PBST (10 mM sodium phosphate pH 7.4, 150 mM NaCl, 0.01% Tween-20) as reaction buffers. STAT1 protein was buffer-exchanged into reaction buffer supplemented with 2% DMSO on a NAP-5 deslating column (Cytiva #17085301) and its concentration was adjusted to 10 or 20 µM. Peptides were diluted in reaction buffer from DMSO stocks to a matching 2% DMSO concentration. Peptide concentrations were adjusted to approximately 100-200 µM. ITC experiments were performed with STAT1 in the cell and peptides in the syringe at 25°C, with 750 RPM stirring, DP = 4 µcal/sec. 18 injections of 2 µl were performed over a 50 min titration. Thermogram integration and isotherm fitting were performed using the PEAQ ITC analysis software (Malvern Panalytical). Isotherms were fitted using a 1:1 binding model. Due to challenges in determining peptide concentrations with high accuracy, protein concentrations and stoichiometry (N=1.0) were kept constant during isotherm fitting, whereas peptide concentrations were allowed to vary. Fixing a single reactant concentration during fitting is acceptable given the sufficiently high C-values and isotherm curvature observed in the experiments.[3] As the STAT1 protein is highly stable at room temperature, decrease in activity over time was not observed. Offset subtraction was implemented to remove injection heats.

Grating coupled interferometry (GCI)

GCI experiments were performed on a Creoptix WaveDELTA instrument (Malvern Panalytical). Full-length STAT1 or STAT3 were immobilised on a 4PCH chip (Malvern Panalytical #4PCH-03) to a density 2000-8000 pg/mm^2 using EDC/NHS activation followed by amine coupling. Briefly, proteins (500 nM) were applied onto an activated chip in 10 mM MES pH 6.5, 20 mM NaCl, after which the chip surface was passivated with 1M ethanolamine, pH 8.0. The kinetic measurements were performed using 20 mM HEPES pH 7.5, 300 mM NaCl, 0.01% Tween-20, 0.0005% DMSO as running buffer. Peptides were tested at 0.5 or 10 µM concentration using the waveRAPID kinetics experiment with the tight binder or intermediate binder mode, respectively.[4] Sensorgrams were fitted in the waveControl software (Malven Panalytical) using the 1:1 kinetic model.

Protein X-ray crystallography

Crystallisation screening of STAT1:peptide complexes was done in 96-well MRC plates using the sitting-drop vapor diffusion technique and a variety of commercial crystallisation screens. Each drop contained 1 µl protein and 1 µl screening condition. Plates were stored at 20 °C. Crystals were cryo-protected by adding 1:4 volume of 50% glycerol to the drop and flash-frozen in liquid nitrogen. Native datasets were collected at Diamond Light Source beamline i04 using the unattended data collection (UDC) mode. Molecular replacement phasing method was used with STAT1:018 complex (PDB: 7NUF) as a search model. Molecular replacement was done with Phaser.[5] Manual refinement was done in Coot[6] and automated refinement with phenix.refine.[7] Crystallisation conditions, as well as data collection and refinement statistics, are provided in **Table S1**. The coordinates have been deposited in the Protein Data Bank under accession codes 9IFX and 9IGA.

Molecular dynamics simulations

The systems used in MD simulations were based on the 018:STAT1 core fragment complex crystal structure (PDB: 7NUF), which corresponds to the linear peptide **1**. The structure of peptide **4** was manually modelled in Coot. Structures were processed using the “Protein Preparation Wizard” tool in Maestro (Schrödinger Release 2024-4) with default settings: missing atoms, sidechains, and loops were modelled, protein protonation states were assigned with PROPKA at pH 7.2, zero bond order constraints to neighbouring atoms were assigned, and the hydrogen bonding network was optimised.[8] To relieve local clashes, a restrained minimisation was performed with a 0.3 Å heavy-atom RMSD displacement cut-

off, below which the minimisation was terminated. For the systems that contain only the peptide, STAT1 atoms were deleted.

Molecular systems for the MD simulations were prepared using the System Builder tool in Maestro. Peptides or peptide-STAT1 complexes were solvated in a triclinic solvent box using the Tip3P solvent model, and the system size was set to extend 10 Å beyond the protein in all directions.[9] Na^+ and Cl^- counterions were added to neutralise systems.

The molecular dynamics simulations were carried out using the Desmond simulation package (Schrödinger Release 2024-4).[10] The prepared systems were relaxed and equilibrated using the default Desmond relaxation protocol. System equilibration was performed in five steps: (i) 100 ps at 10 K with Brownian dynamics, NVT ensemble, solute heavy atom restraints and small (1 fs) timestep; (ii) 12 ps at 10 K with Berendsen thermostat, NVT ensemble, solute heavy atom restraints and small timestep; (iii) 12 ps at 10 K with Berendsen, NPT ensemble, solute heavy atom restraints, increase to default timestep; (iv) 24 ps at 300 K with Berendsen, NPT ensemble, solute heavy atom restraints; and (v) 240 ps at 300 K with Berendsen, NPT ensemble and no restraints.

The production MD simulations were carried out at constant pressure (1.0 bar) maintained using a Martyna–Tobias–Klein barostat[11] and at constant temperature (300 K) maintained using a Nose–Hoover thermostat.[12] The pressure and temperature control used a relaxation time of 2.0 and 1.0 ps, respectively. The Coulombic interaction cutoff was set to 9.0 Å. The OPLS4 force field[13] was used for all simulations. All simulations used a RESPA integrator[14] with a 2.0 fs time step. The total simulation length for each system was 1000 ns. The trajectories were saved at a 200 ps interval for analysis (a total of 5000 frames). The obtained trajectories were analysed using the Simulation Interaction Diagram tools implemented in the Desmond molecular dynamics package.

Fluorescence polarisation (FP) assay

FP measurements were performed in black, flat-bottom, low-volume 384-well plates with low-binding surfaces (Corning #3820). FP reactions were set up in 20 mM Tris pH 8.0, 300 mM NaCl, 0.1% Tween-20, 0.5 mM TCEP, 2.5% DMSO in 40 µl volume at room temperature. All competition reactions contained 10 nM fluorescein-conjugated, Tyrosine-phosphorylated peptide probe containing to the IFNGR1 STAT1 docking site (5Flu-GTSFG<u>pYDKPH</u>VLV-NH2). STAT1 was kept at 1.5 µM, which corresponds to approximately 80% saturation of the probe. Peptides were first serially-diluted in DMSO and then diluted with the reaction buffer, followed by the addition of STAT1 and the fluorescent probe. As controls, reactions containing only the fluorescent probe were included for gain adjustment. FP measurements were

recorded on a Clariostar Plus plate reader (BMG Labtech) using 482-16/504/530-40 nm filters for excitation, dichroic and emission, respectively. To determine IC_{50} values, FP data were normalised and fitted using a four parameter logistic equation in Prism software (GraphPad).

Results and Discussion

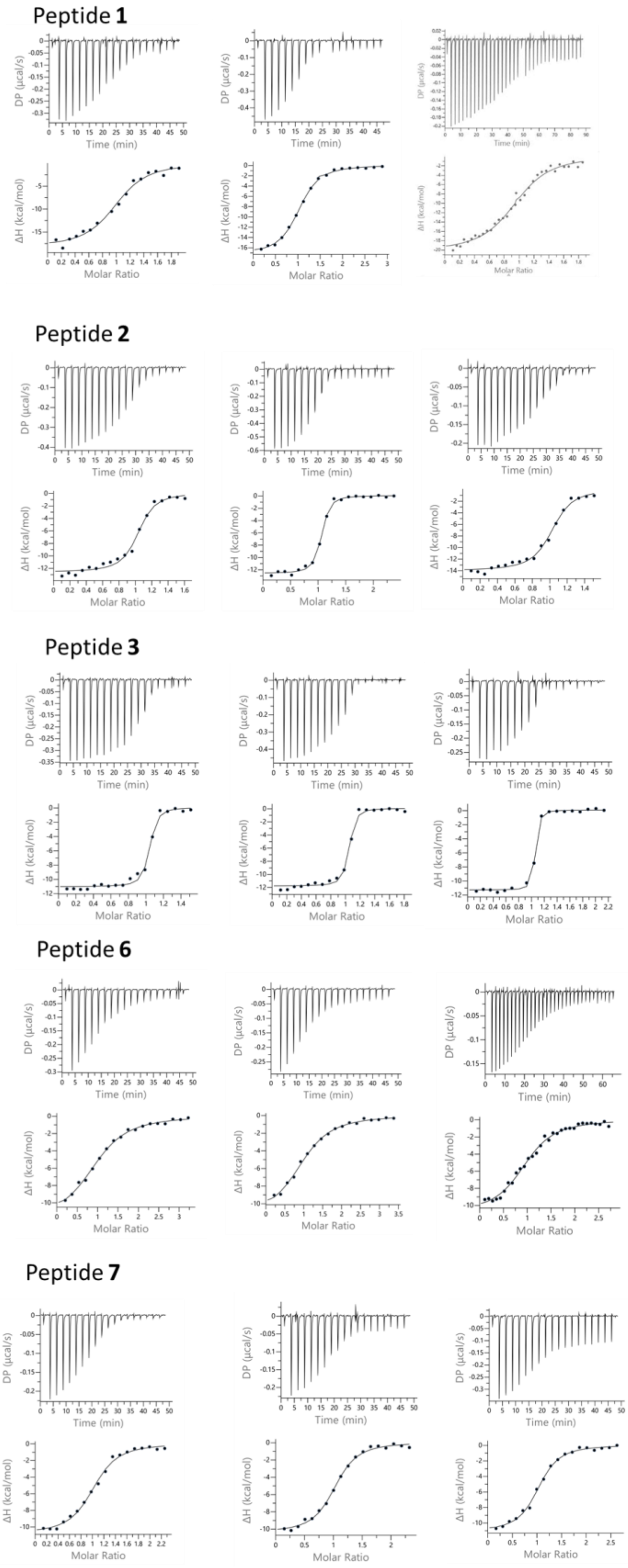

Figure S1. ITC thermograms and isotherms of peptides binding to STAT1 in TBST buffer system (50 mM Tris pH 8.0, 300 mM NaCl, 0.01% Tween-20). The fitted parameters are provided in **Table S1**.

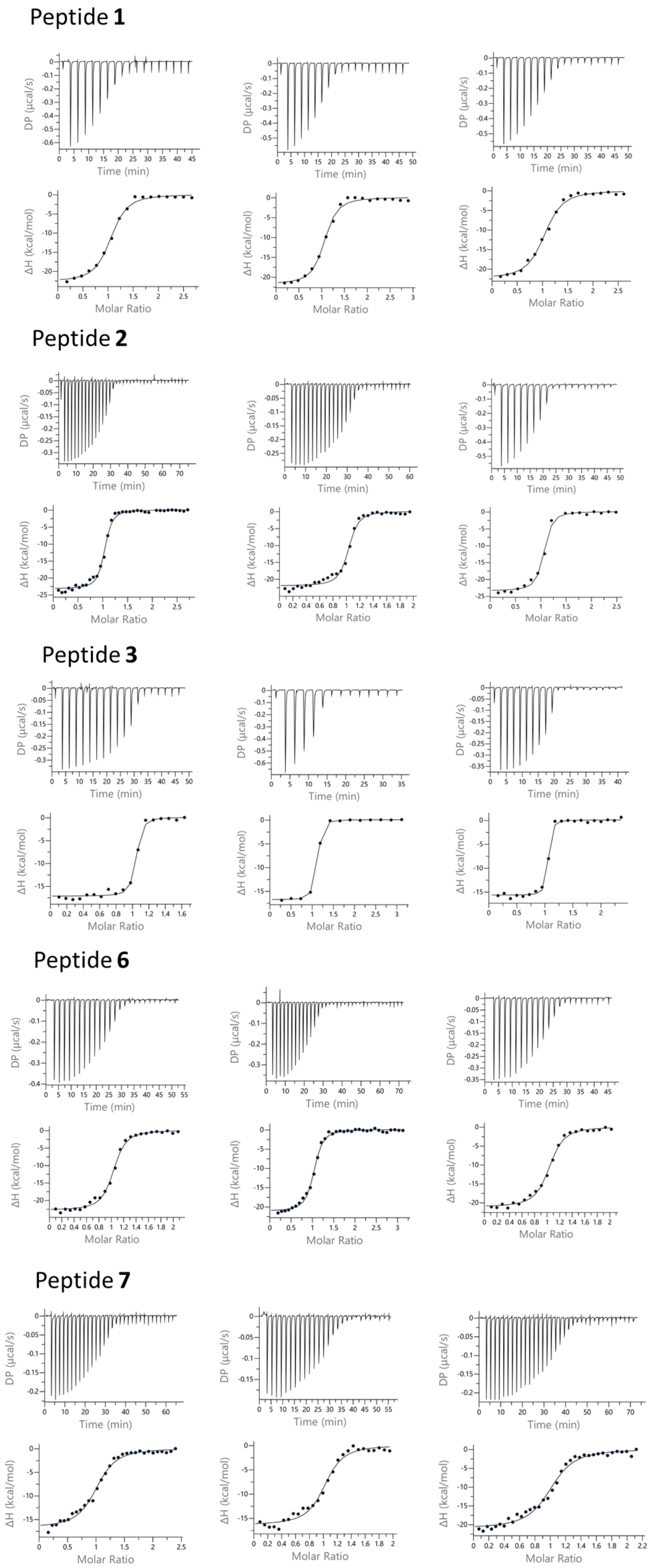

Figure S2. ITC thermograms and isotherms of peptides binding to STAT1 in PBST buffer system (10 mM sodium phosphate pH 7.4, 150 mM NaCl, 0.01% Tween-20). The fitted parameters are shown in **Table S1**.

Peptide	Buffer system	[Syringe], µM	[Cell], µM	**K_D**, nM	**ΔH** (kcal/mol)	**ΔG** (kcal/mol)	**-TΔS** (kcal/mol)	Offset (kcal/mol)
**1**	TBST	99	10	548	-18.1	-8.54	9.86	-0.236
**1**	TBST	184	10	511	-17.3	-8.58	8.75	-0.390
**1**	TBST	49	10	541	-20.3	-8.55	11.7	-0.337
**2**	TBST	99	10	350	-17.2	-8.81	8.44	-0.244
**2**	TBST	87	10	310	-17.8	-8.87	8.91	0.537
**3**	TBST	167	20	202	-12.7	-9.14	3.53	-0.012
**3**	TBST	246	20	101	-12.7	-9.54	3.12	-0.447
**3**	TBST	78	10	131	-14.0	-9.40	4.65	-0.161
**4**	TBST	157	20	33	-11.1	-10.2	0.909	-0.107
**4**	TBST	199	10	18	-10.6	-10.6	0.050	-0.121
**4**	TBST	111	10	7	-11.5	-11.1	0.239	-0.489
**7**	TBST	175	10	1870	-11.5	-7.83	3.67	-0.378
**7**	TBST	167	10	1990	-12.1	-7.78	4.28	-0.456
**7**	TBST	165	10	1330	-11.7	-8.02	3.66	-0.474
**8**	TBST	136	10	455	-11.2	-8.65	2.52	-1.94
**8**	TBST	120	10	385	-10.5	-8.75	1.72	-0.439
**8**	TBST	117	10	469	-10.9	-8.64	2.24	-0.152
**1**	PBST	147	10	255	-22.8	-9.00	13.8	-0.498
**1**	PBST	150	10	191	-21.8	-9.17	12.7	-1.03
**1**	PBST	136	10	314	-22.6	-8.87	13.7	-0.679
**2**	PBST	103	10	114	-24.3	-9.47	14.0	0.494
**2**	PBST	99	10	148	-24.2	-9.32	14.9	0.600
**3**	PBST	129	10	59	-23.5	-9.86	13.6	-0.789
**3**	PBST	141	10	55	-23.3	-9.91	13.4	-0.463
**3**	PBST	128	10	52	-22.1	-9.93	12.2	-0.291
**4**	PBST	93	11	12	-17.3	-10.8	6.48	-0.811
**4**	PBST	227	10	8	-15.7	-11.0	4.69	-0.490
**4**	PBST	228	10	21	-16.9	-10.5	6.38	-0.765
**7**	PBST	160	10	97.7	-22.9	-9.57	13.3	0.159
**7**	PBST	168	10	108	-21.2	-9.51	11.7	-0.322
**7**	PBST	170	10	138	-21.1	-9.37	11.7	0.010
**8**	PBST	142	10	315	-16.8	-8.87	7.96	-0.066
**8**	PBST	138	10	180	-16.5	-9.20	7.25	-0.063

Table S1. Fitted ITC parameters for peptide binding to STAT1.

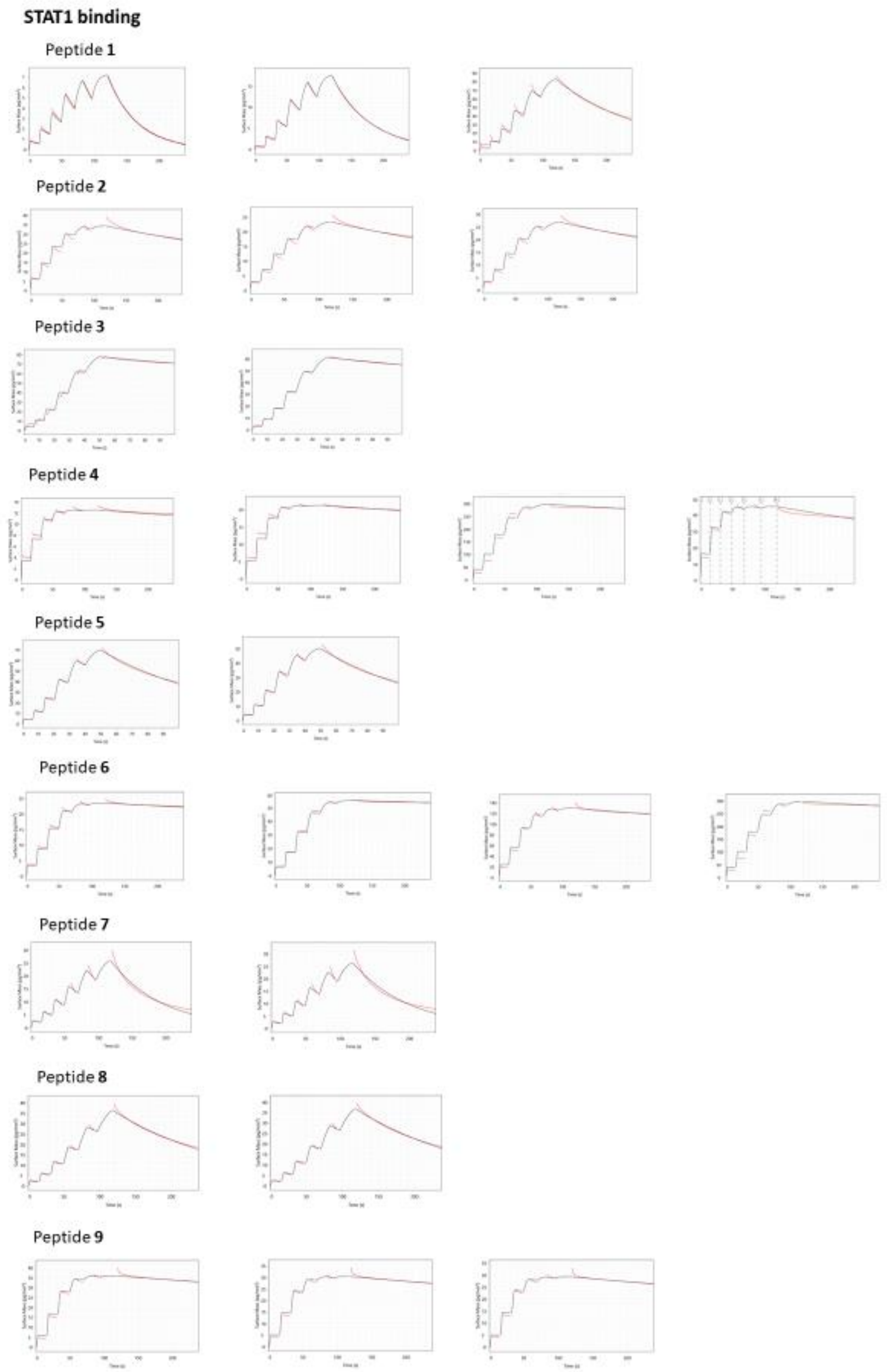

Figure S3. GCI sensorgrams depicting RAPID kinetics measurements and fitted kinetic models for peptide binding to full-length STAT1 immobilised on a polycarboxylate hydrogel chip (4PCH). 500 nM peptides were applied using the strong binders pulse sequence.

Peptide **1**

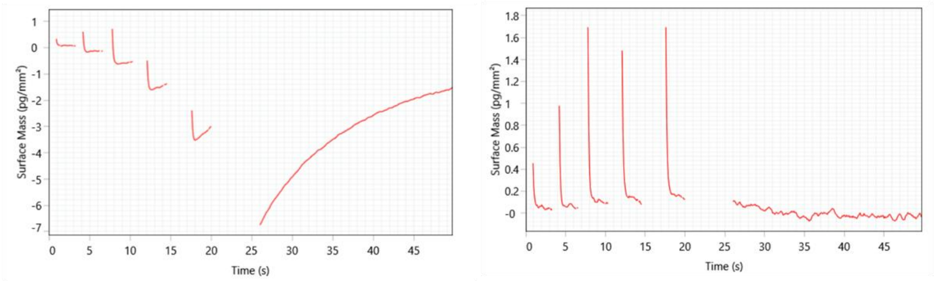

Peptide **3**

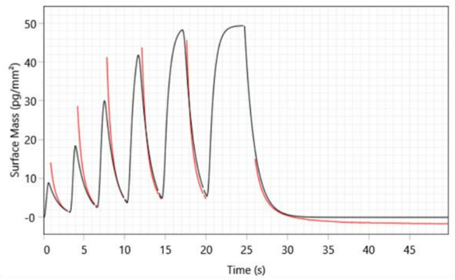

Peptide **4**

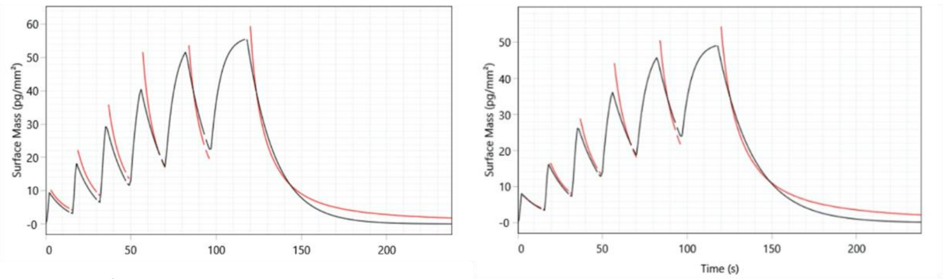

Peptide **5**

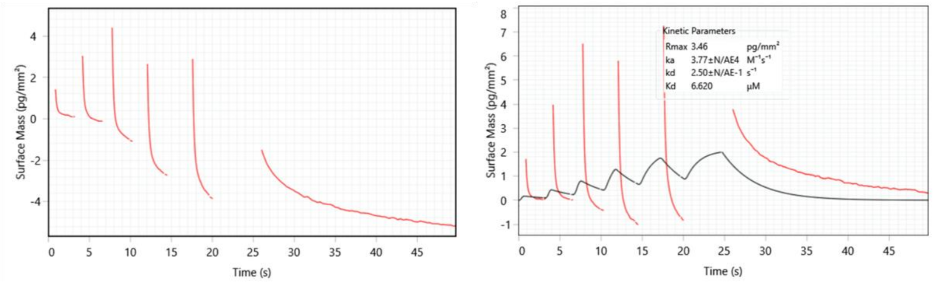

Peptide **6**

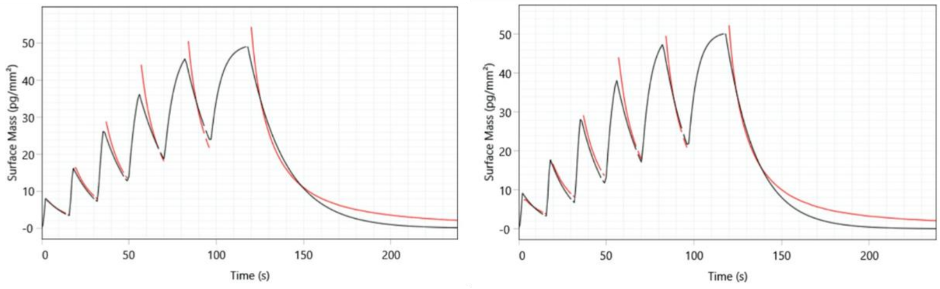

Figure S4. GCI sensorgrams depicting RAPID kinetics measurements and fitted kinetic models for peptide binding to full-length STAT3 immobilised on a polycarboxylate hydrogel chip (4PCH). Peptides were applied at 0.5 or 10 µM concentration using the medium or tight binders pulse sequence. The peptides that show negligible Rmax values at 10 µM analyte concentration were classified as non-binding.

Analyte	[Analyte], μM	Ligand	K_D, nM	k_a, $M^{-1}s^{-1}$	k_d, s^{-1}	R_{max}, pg*mm^{-2}
**1**	0.5	STAT1	91.9	2.41E+5	2.21E-2	8.65
**1**	0.5	STAT1	79.22	2.17E+5	1.72E-2	20.85
**1**	0.5	STAT1	86.31	2.62E+5	2.26E-2	11.15
**2**	0.5	STAT1	9.76	2.16E+5	2.11E-3	27.68
**2**	0.5	STAT1	10.50	2.13E+5	2.24E-3	24.15
**2**	0.5	STAT1	6.401	3.21E+5	2.05E-3	34.96
**3**	0.5	STAT1	7.16	2.61E+5	1.87E-3	85.97
**3**	0.5	STAT1	7.87	2.76E+5	2.17E-3	67.04
**4**	0.5	STAT1	1.08	6.58E+5	7.09E-4	12.56
**4**	0.5	STAT1	1.01	5.94E+5	6.01E-4	21.32
**4**	0.5	STAT1	1.76	2.77E+5	4.89E-4	299.99
**4**	0.5	STAT1	1.77	8.65E+5	1.53E-3	45.31
**5**	0.5	STAT1	35.00	3.51E+5	1.23E-2	78.11
**5**	0.5	STAT1	28.89	4.57E+5	1.32E-2	54.33
**6**	0.5	STAT1	1.31	3.77E+5	4.94E-4	23.60
**6**	0.5	STAT1	0.88	3.04E+5	2.68E-4	55.61
**6**	0.5	STAT1	3.06	1.86E+5	5.72E-4	141.06
**6**	0.5	STAT1	2.03	3.84E+5	7.81E-4	130.91
**7**	0.5	STAT1	101.6	1.29E+5	1.31E-2	33.57
**7**	0.5	STAT1	94.70	1.31E+5	1.24E-2	33.75
**7**	0.5	STAT1	94.52	1.37E+5	1.29E-2	33.40
**8**	0.5	STAT1	74.83	8.70E+4	6.04E-3	50.22
**8**	0.5	STAT1	76.02	8.00E+4	6.08E-3	49.88
**9**	0.5	STAT1	1.52	5.87E+5	8.91E-4	29.39
**9**	0.5	STAT1	1.53	5.70E+5	8.69E-4	30.56
**9**	0.5	STAT1	1.38	5.40E+5	7.46E-4	32.90
**1**	10	STAT3	n.b.	-	-	-
**1**	10	STAT3	n.b.	-	-	-
**3**	10	STAT3	9617	8.39E+4	8.07E-1	96.94
**3**	10	STAT3	8976	8.10E+4	7.27E-1	76.47
**4**	0.5	STAT3	240	2.57E+5	6.18E-2	81.21
**4**	0.5	STAT3	255	2.39E+5	6.11E-2	85.25
**5**	10	STAT3	n.b.	-	-	-
**5**	10	STAT3	n.b.	-	-	-
**6**	0.5	STAT3	187	2.52E+5	4.73E-2	68.60
**6**	0.5	STAT3	204	2.80E+5	5.71E-2	71.44

n.b. – no binding observed

Table S2. Kinetic parameters for the fitted GCI measurements.

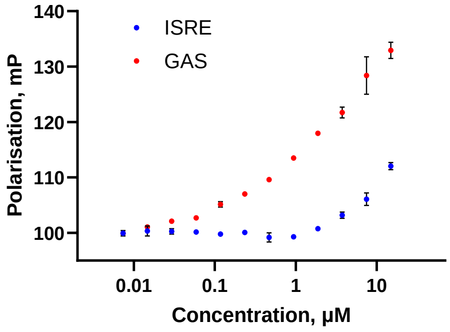

Figure S5. Fluorescence polarisation (FP) measurements for the direct titration of Tyr-phosphorylated STAT1 and FAM-labelled double-stranded oligonucleotides corresponding to the ISRE and GAS sequences.

X-ray crystallographic data collection and refinement

	9IFX	9IGA
**Protein condition**	20 mM Tris pH 8.0, 300 mM NaCl, 0.5 mM TCEP-NaOH	20 mM Tris pH 8.0, 300 mM NaCl, 0.5 mM TCEP-NaOH
**Crystallisation condition**	4% v/v TacsimateTM pH 5.0, 12% w/v PEG 3350	0.1 M Bis-Tris pH 5.5, 0.2 M Ammonium acetate, 25 % w/v PEG 3350
**Protein:well solution (nl:nl)**	1000:1000	1000:1000
**PDB code**	9IFX	9IGA
**Data collection and processing**		
Beamline	Diamond i04	Diamond i04
Wavelength (Å)	0.9537	0.9537
Space group	P 42	P 2 21 21
a, b, c (Å)	161.14 161.14 38.48	35.66 117.27 130.28
α, β, γ (°)	90.00 90.00 90.00	90.00 90.00 90.00
Resolution range (Å)	3.64 - 113.94 (3.64 - 3.71)	2.80 - 65.14 (2.80 - 2.85)
R_{meas}	0.309 (4.247)	0.451 (2.844)
Completeness (%)	94.3 (97.1)	100 (96.9)
Unique reflections	10932 (538)	14172 (663)
Redundancy	10.6 (10.1)	13.1 (13.3)
<I/σ(I)>	5.7 (1.4)	4.1 (0.6)
CC½	1.0 (0.2)	1.0 (0.7)
**Refinemnt**		
R_{cryst}/R_{free}	0.2284 / 0.2708	0.3081 / 0.3138
Resolution range (Å)	56.97 - 3.64	58.64 - 2.802
Reflections in work / test set	10717 (942)	14080 (1326) / 681 (61)
Number of atoms	4465	4202
Mean / Wilson B-factor ($Å^2$)	117.49 / 111.45	60.34 / 56.45
Ramachandran favoured/allowed/outliers (%)	94.75 / 4.88 / 0.38	95.77 / 3.83 / 0.40
RMSD bonds (Å)	0.012	0.011
RMSD angles (°)	1.37	1.35

Table S3. X-ray crystallographic data collection and refinement.

Peptide analytical data

Peptide **1**

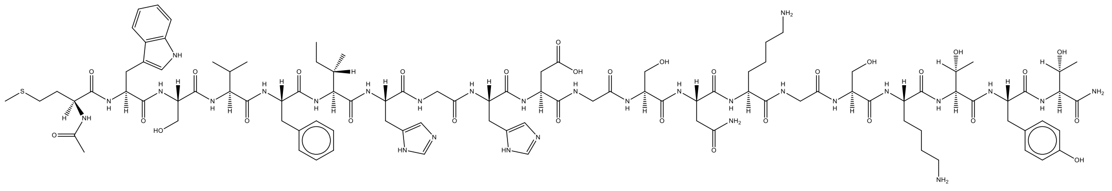

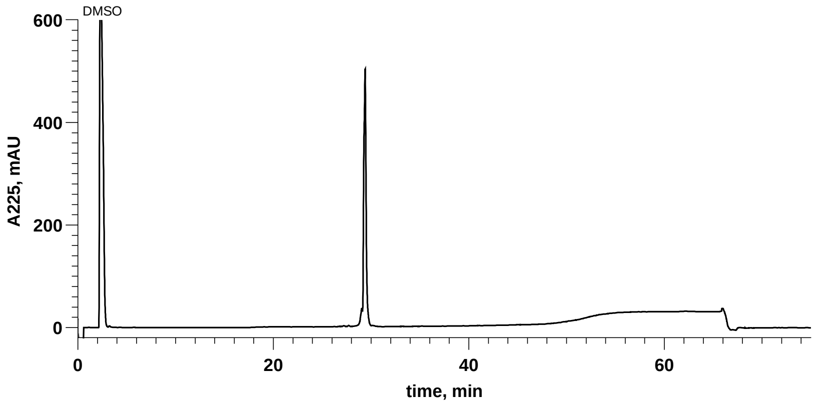

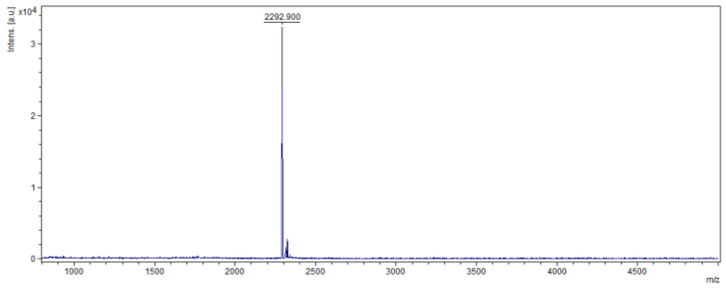

	$[M+H]^{1+}$
m/z calc	2293.078
m/z obs	2292.900
Accuracy, ppm	77

Peptide **2**

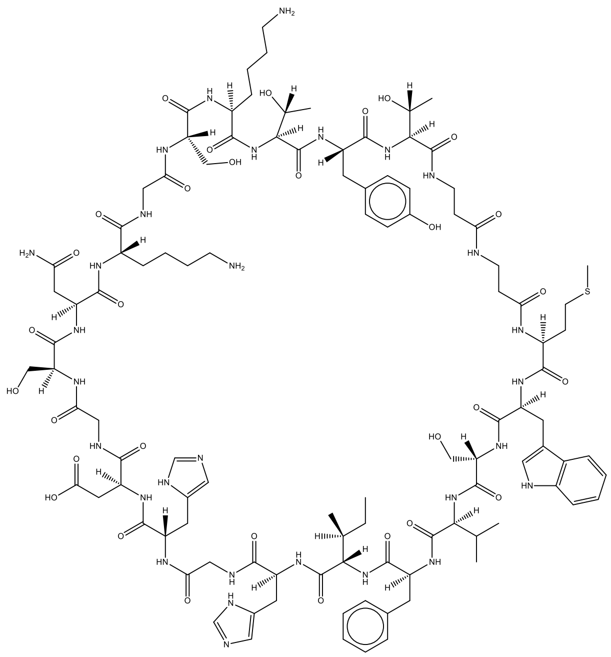

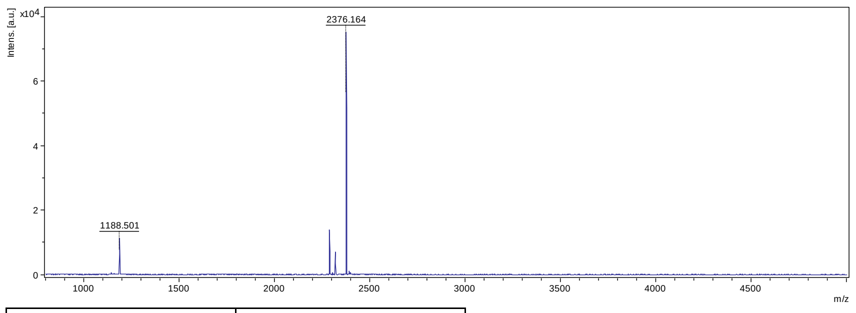

	$[M+H]^{1+}$
m/z calc	2376.115
m/z obs	2376.164
Accuracy, ppm	20

Peptide **3**

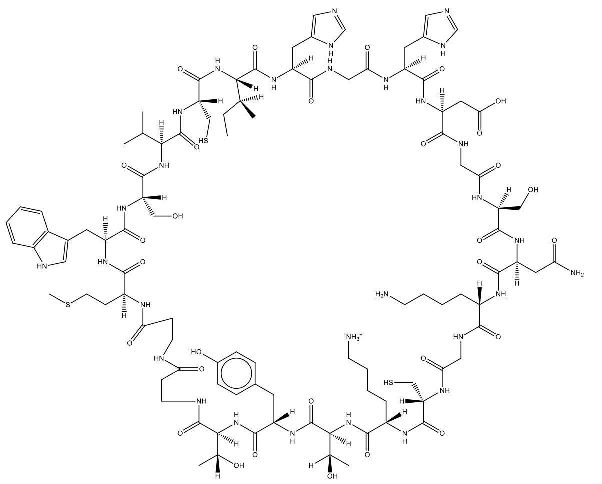

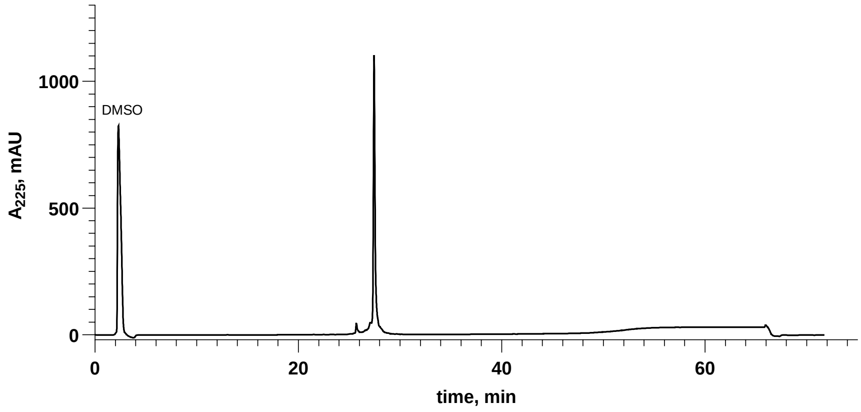

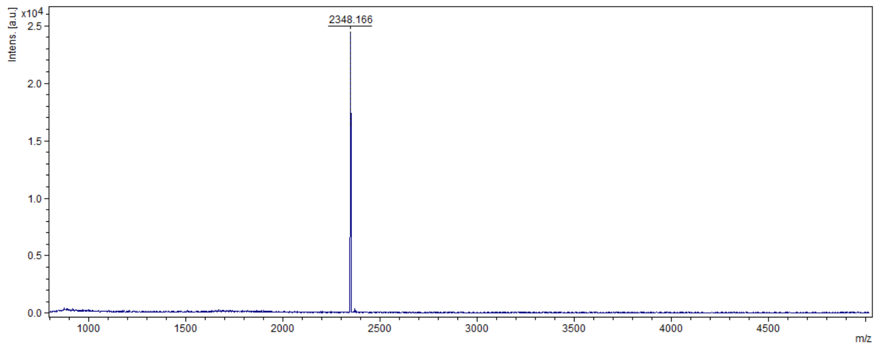

	[M+H]$^{1+}$
m/z calc	2348.040
m/z obs	2348.166
Accuracy, ppm	53

Peptide **4**

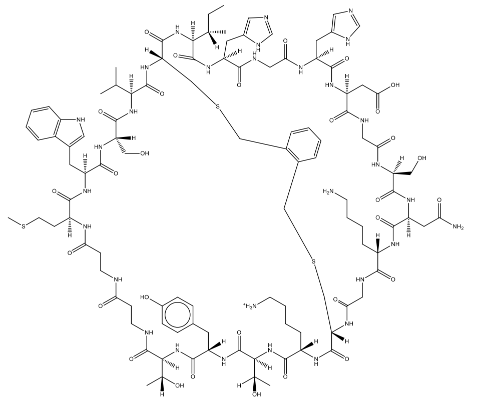

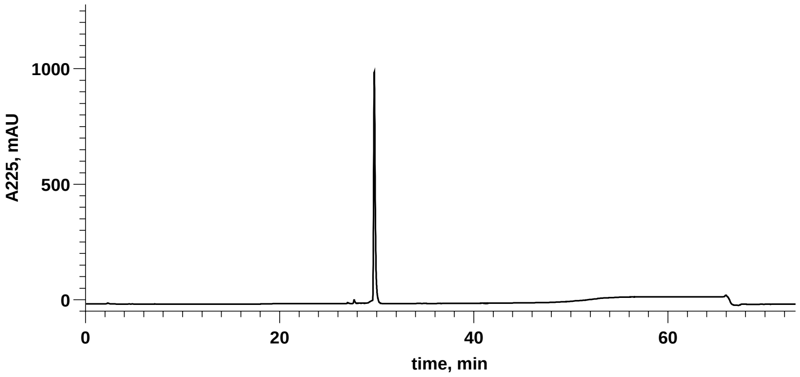

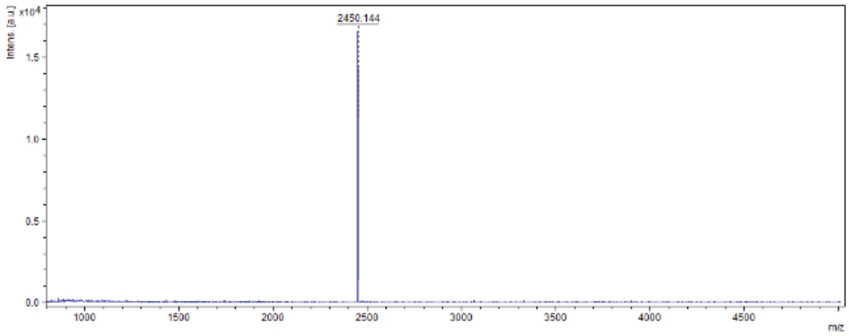

	$[M+H]^{1+}$
m/z calc	2450.084
m/z obs	2450.144
Accuracy, ppm	24

Peptide **5**

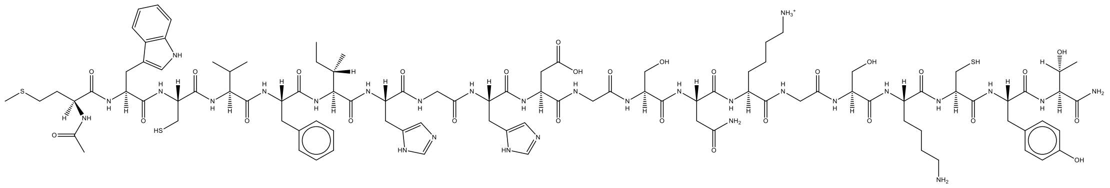

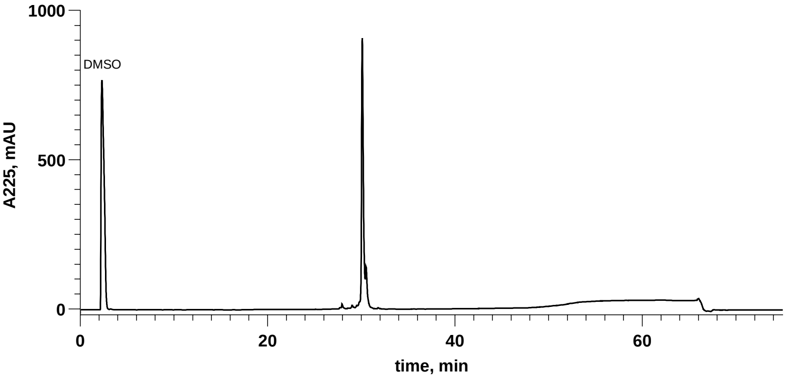

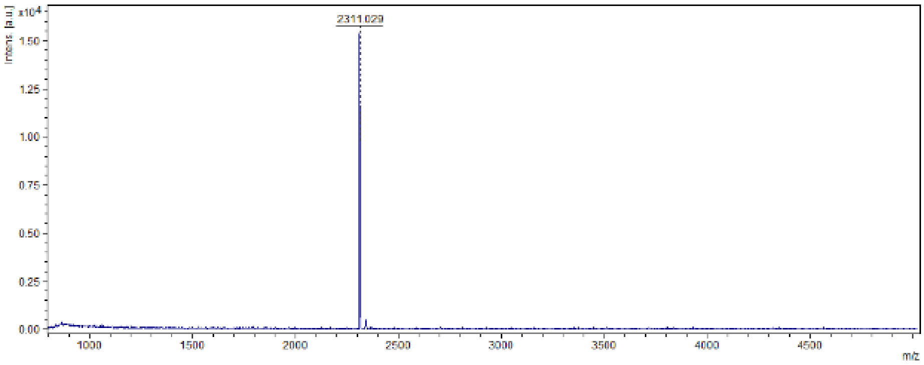

	$[M+H]^{1+}$
m/z calc	2311.021
m/z obs	2311.029
Accuracy, ppm	3

Peptide **6**

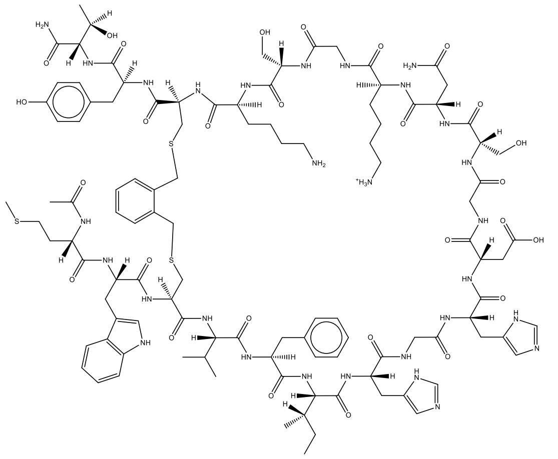

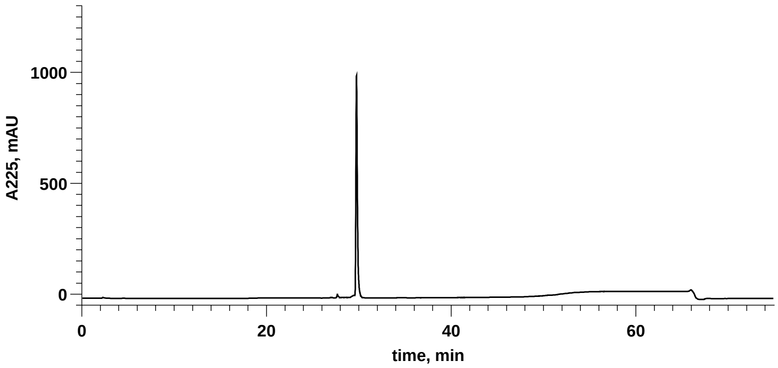

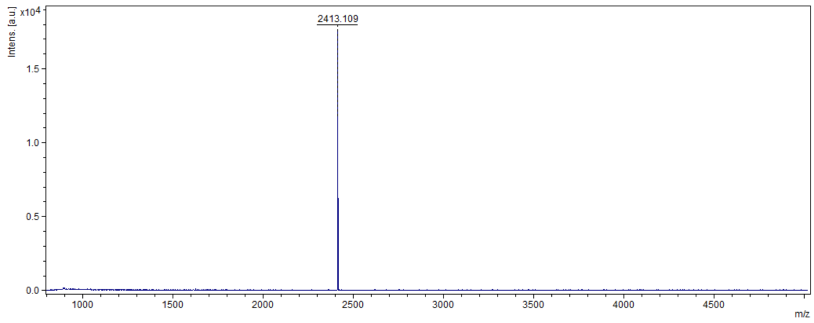

	$[M+H]^{1+}$
m/z calc	2413.067
m/z obs	2413.109
Accuracy, ppm	17

Peptide **7**

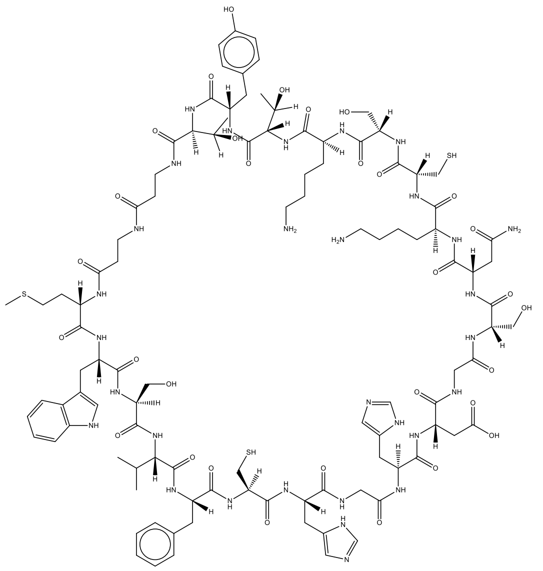

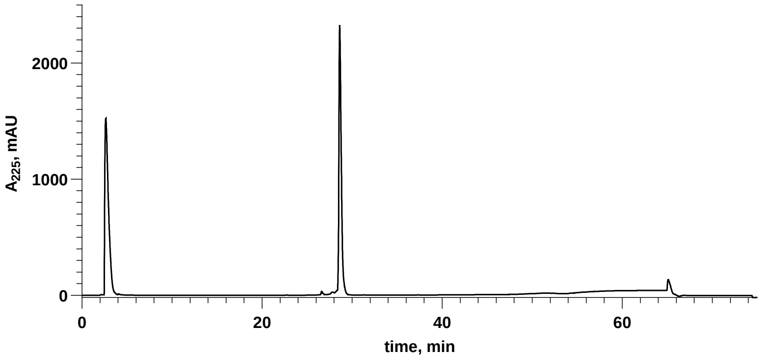

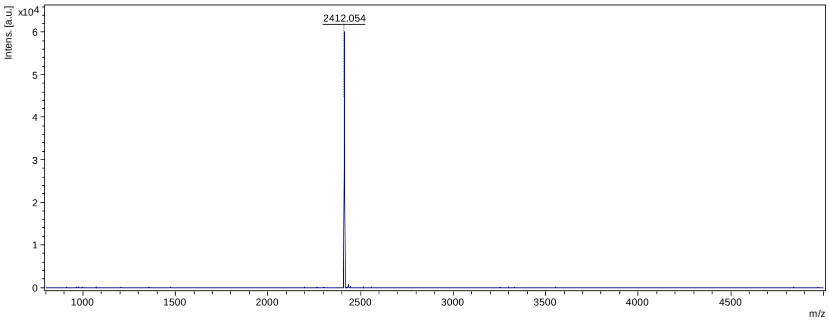

	$[M+H]^{1+}$
m/z calc	2412.025
m/z obs	2412.054
Accuracy, ppm	12

Peptide **8**

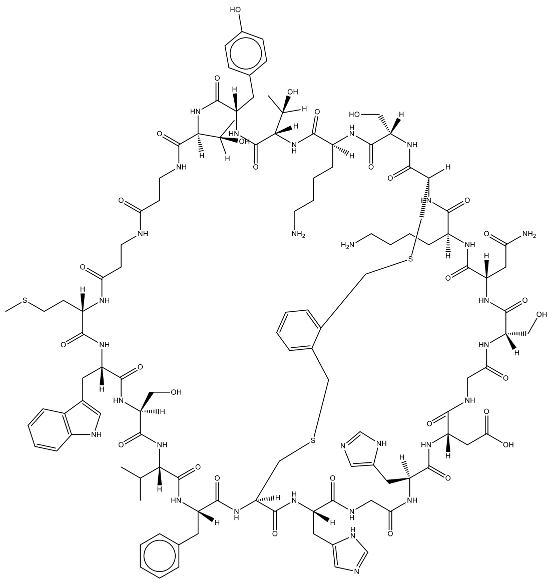

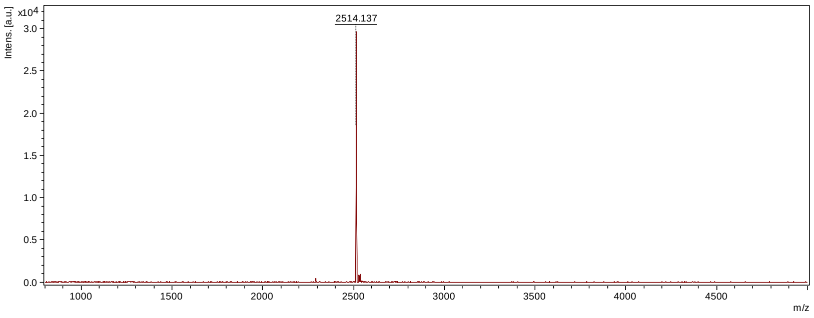

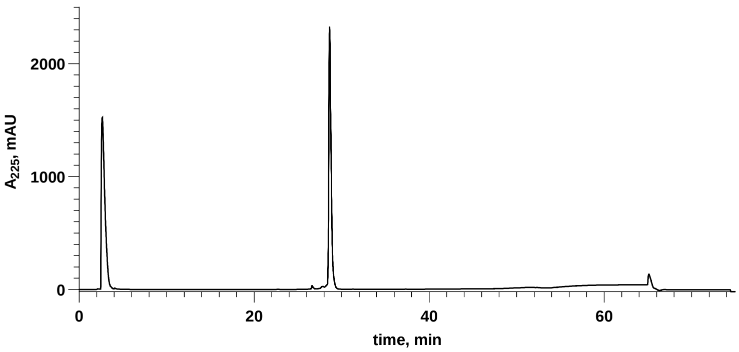

	$[M+H]^{1+}$
m/z calc	2514.075
m/z obs	2514.137
Accuracy, ppm	24

Peptide **9**

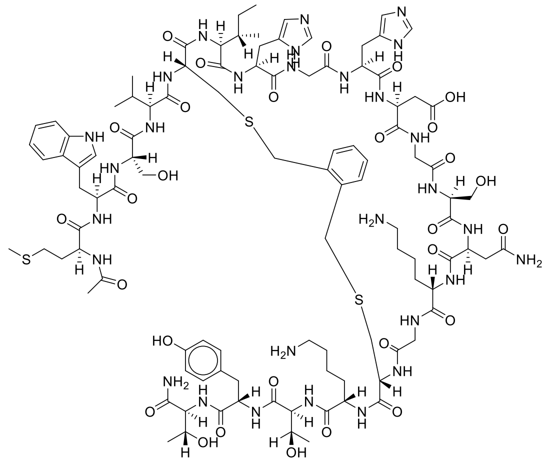

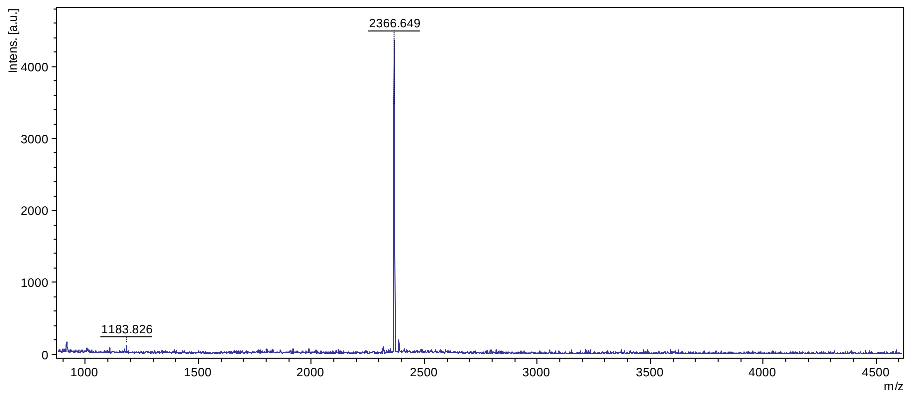

	[M+H]$^{1+}$
m/z calc	2367.04
m/z obs	2366.65
Accuracy, ppm	1464

STAT3 synthetic gene sequence

GAAAACCTGTACTTCCAGGGAAGTGCCCAGTGGAATCAGCTGCAGCAACTGGATACGCGTTATTTAGAACAGCTGCATCAACTTTA
TTCAGATTCTTTTCCGATGGAACTTCGACAGTTCTTAGCGCCGTGGATCGAGTCTCAGGACTGGGCATATGCTGCTTCCAAGGAAT
CACATGCCACGCTGGTATTTCATAACCTGCTGGGTGAAATCGATCAACAGTATTCGCGTTTTTTACAAGAAAGCAATGTTCTGTAC
CAGCATAATTTGCGCAGAATAAAACAATTTCTGCAGTCACGTTATTTAGAAAAACCAATGGAGATAGCAAGAATAGTGGCGCGCTG
TCTGTGGGAGGAGTCACGTCTTCTTCAAACCGCAGCAACCGCAGCGCAGCAGGGTGGACAGGCAAATCACCCAACGGCAGCCGTAG
TCACGGAAAAACAACAGATGCTGGAGCAACATCTGCAGGACGTCAGAAAACGTGTCCAGGATTTAGAGCAGAAAATGAAAGTTGTT
GAGAACCTCCAGGATGATTTTGACTTTAACTACAAAACGCTCAAATCGCAGGGTGACATGCAGGACCTCAATGGAAATAACCAAAG
CGTTACCCGACAGAAGATGCAACAGCTCGAGCAGATGCTGACTGCTTTAGATCAGATGCGTCGCTCTATCGTCAGTGAATTAGCTG
GTCTTCTGTCTGCAATGGAGTATGTTCAGAAGACACTGACAGACGAAGAGCTGGCAGACTGGAAGCGTCGTCAGCAGATCGCATGT
ATCGGAGGCCCTCCAAATATCTGTCTTGATCGCCTTGAAAACTGGATCACAAGCCTTGCAGAATCACAACTTCAGACCCGTCAGCA
GATCAAGAAACTTGAAGAATTACAGCAAAAAGTTTCGTATAAAGGAGATCCGATTGTTCAGCACAGACCAATGTTAGAGGAGCGCA
TTGTCGAACTGTTTAGAAATCTTATGAAATCTGCATTTGTCGTTGAAAGACAGCCATGCATGCCGATGCATCCGGATCGCCCGCTG
GTTATTAAGACAGGAGTACAATTCACGACCAAAGTTCGACTGCTCGTGAAATTTCCGGAATTAAACTATCAGCTTAAAATAAAAGT
GTGCATTGATAAAGATAGTGGTGACGTGGCGGCATTGAGAGGAAGCAGAAAATTCAATATACTGGGTACGAATACCAAGGTGATGA
ACATGGAGGAATCGAACAACGGATCACTGTCGGCTGAGTTTAAACACTTAACTCTCCGTGAACAGCGTTGCGGGAATGGTGGTAGA
GCGAATTGCGATGCGTCCCTGATCGTTACAGAAGAGTTACATCTCATTACATTTGAAACGGAGGTGTACCATCAGGGCTTGAAGAT
CGATCTGGAGACGCATAGCCTGCCGGTAGTCGTGATTAGTAACATTTGCCAAATGCCAAATGCCTGGGCTTCTATTCTGTGGTACA
ACATGCTTACCAATAATCCCAAAAACGTTAATTTTTTTACCAAGCCTCCGATTGGTACTTGGGACCAGGTCGCTGAGGTATTGTCA
TGGCAATTTAGTTCGACAACAAAAAGAGGCCTGAGCATAGAACAGTTAACTACGTTAGCCGAGAAATTGCTGGGGCCGGGAGTGAA
TTACTCCGGGTGTCAGATCACCTGGGCCAAATTTTGCAAGGAAAACATGGCAGGAAAAGGCTTCTCATTTTGGGTATGGTTAGATA
ACATAATCGATTTAGTAAAAAAATATATTCTCGCATTGTGGAATGAAGGATATATTATGGGGTTTATCAGTAAAGAACGGGAAAGA
GCGATCCTGAGTACCAAACCGCCGGGAACTTTCCTGCTGCGCTTTTCAGAATCTTCTAAAGAAGGCGGGGTTACTTTTACATGGGT
CGAGAAAGATATCAGTGGGAAGACGCAGATCCAGAGTGTCGAACCATATACAAAGCAACAGTTAAATAACATGTCTTTCGCAGAGA
TCATCATGGGCTACAAAATAATGGATGCAACAAACATTTTGGTAAGTCCGTTAGTGTATTTATACCCGGATATACCGAAAGAAGAA
GCTTTCGGTAAGTACTGCCGGCCTGAGTCACAGGAACATCCCGAAGCCGACCCCGGTTCTGCGGCCCCTTACCTTAAAACAAAATT
CATATGCGTGACACCGACGACATGCTCGAACACCATAGATCTGCCGATGTCCCCTCGTACCTTGGATTCACTGATGCAATTTGGGA
ACAATGGAGAGGGGGCAGAACCGTCAGCTGGTGGGCAGTTTGAGAGCTTGACGTTTGATATGGAGTTAACCTCTGAATGTGCAACA
TCTCCAATGTAAGCTTGAGTATTCTATAG

Bibliography

[1] E. Gasteiger, C. Hoogland, A. Gattiker, S. Duvaud, M. R. Wilkins, R. D. Appel, A. Bairoch, in *The Proteomics Protocols Handbook* (Ed.: J.M. Walker), Humana Press, Totowa, NJ, **2005**, pp. 571–607.

[2] C. Talbot-Cooper, T. Pantelejevs, J. P. Shannon, C. R. Cherry, M. T. Au, M. Hyvönen, H. D. Hickman, G. L. Smith, *Cell Host & Microbe* **2022**, *30*, 357-372.e11.

[3] S. A. Kantonen, N. M. Henriksen, M. K. Gilson, *Biochimica et Biophysica Acta (BBA) - General Subjects* **2017**, *1861*, 485–498.

[4] Ö. Kartal, F. Andres, M. P. Lai, R. Nehme, K. Cottier, *SLAS Discovery* **2021**, *26*, 995–1003.

[5] A. J. McCoy, R. W. Grosse-Kunstleve, P. D. Adams, M. D. Winn, L. C. Storoni, R. J. Read, *Journal of Applied Crystallography* **2007**, *40*, 658–674.

[6] P. Emsley, B. Lohkamp, W. G. Scott, K. Cowtan, *Acta Crystallogr D Biol Crystallogr* **2010**, *66*, 486–501.

[7] P. V. Afonine, R. W. Grosse-Kunstleve, N. Echols, J. J. Headd, N. W. Moriarty, M. Mustyakimov, T. C. Terwilliger, A. Urzhumtsev, P. H. Zwart, P. D. Adams, *Acta Crystallographica Section D* **2012**, *68*, 352–367.

[8] M. H. M. Olsson, C. R. Søndergaard, M. Rostkowski, J. H. Jensen, *J. Chem. Theory Comput.* **2011**, *7*, 525–537.

[9] P. Mark, L. Nilsson, *J. Phys. Chem. A* **2001**, *105*, 9954–9960.

[10] K. J. Bowers, D. E. Chow, H. Xu, R. O. Dror, M. P. Eastwood, B. A. Gregersen, J. L. Klepeis, I. Kolossvary, M. A. Moraes, F. D. Sacerdoti, J. K. Salmon, Y. Shan, D. E. Shaw, in *SC '06: Proceedings of the 2006 ACM/IEEE Conference on Supercomputing*, **2006**, pp. 43–43.

[11] G. J. Martyna, M. E. Tuckerman, D. J. Tobias, M. L. Klein, *Molecular Physics* **1996**, *87*, 1117–1157.

[12] S. Nosé, *The Journal of Chemical Physics* **1984**, *81*, 511–519.

[13] C. Lu, C. Wu, D. Ghoreishi, W. Chen, L. Wang, W. Damm, G. A. Ross, M. K. Dahlgren, E. Russell, C. D. Von Bargen, R. Abel, R. A. Friesner, E. D. Harder, *J. Chem. Theory Comput.* **2021**, *17*, 4291–4300.

[14] C. I. Bayly, P. Cieplak, W. Cornell, P. A. Kollman, *J. Phys. Chem.* **1993**, *97*, 10269–10280.
